# HIV-1 integrase binding to genomic RNA 5′-UTR induces local structural changes in vitro and in virio

**DOI:** 10.1186/s12977-021-00582-0

**Published:** 2021-11-22

**Authors:** Shuohui Liu, Pratibha C. Koneru, Wen Li, Chathuri Pathirage, Alan N. Engelman, Mamuka Kvaratskhelia, Karin Musier-Forsyth

**Affiliations:** 1 Department of Chemistry and Biochemistry, Centers for RNA Biology and Retroviral Research, The Ohio State University, Columbus, OH USA; 2 Division of Infectious Diseases, School of Medicine, University of Colorado, Aurora, CO USA; 3 Department of Cancer Immunology and Virology, Dana-Farber Cancer Institute, Boston, MA USA; 4 Department of Medicine, Harvard Medical School, Boston, MA USA

**Keywords:** HIV-1, Nucleocapsid, Integrase, XL-SHAPE, 5′-UTR, RNA binding, RNA secondary structure, ALLINI

## Abstract

**Background:**

During HIV-1 maturation, Gag and Gag-Pol polyproteins are proteolytically cleaved and the capsid protein polymerizes to form the honeycomb capsid lattice. HIV-1 integrase (IN) binds the viral genomic RNA (gRNA) and impairment of IN-gRNA binding leads to mis-localization of the nucleocapsid protein (NC)-condensed viral ribonucleoprotein complex outside the capsid core. IN and NC were previously demonstrated to bind to the gRNA in an orthogonal manner in virio; however, the effect of IN binding alone or simultaneous binding of both proteins on gRNA structure is not yet well understood.

**Results:**

Using crosslinking-coupled selective 2′-hydroxyl acylation analyzed by primer extension (XL-SHAPE), we characterized the interaction of IN and NC with the HIV-1 gRNA 5′-untranslated region (5′-UTR). NC preferentially bound to the packaging signal (Psi) and a UG-rich region in U5, irrespective of the presence of IN. IN alone also bound to Psi but pre-incubation with NC largely abolished this interaction. In contrast, IN specifically bound to and affected the nucleotide (nt) dynamics of the apical loop of the transactivation response element (TAR) and the polyA hairpin even in the presence of NC. SHAPE probing of the 5′-UTR RNA in virions produced from allosteric IN inhibitor (ALLINI)-treated cells revealed that while the global secondary structure of the 5′-UTR remained unaltered, the inhibitor treatment induced local reactivity differences, including changes in the apical loop of TAR that are consistent with the in vitro results.

**Conclusions:**

Overall, the binding interactions of NC and IN with the 5′-UTR are largely orthogonal in vitro*.* This study, together with previous probing experiments, suggests that IN and NC binding in vitro and in virio lead to only local structural changes in the regions of the 5′-UTR probed here. Accordingly, disruption of IN-gRNA binding by ALLINI treatment results in local rather than global secondary structure changes of the 5′-UTR in eccentric virus particles.

**Graphical Abstract:**

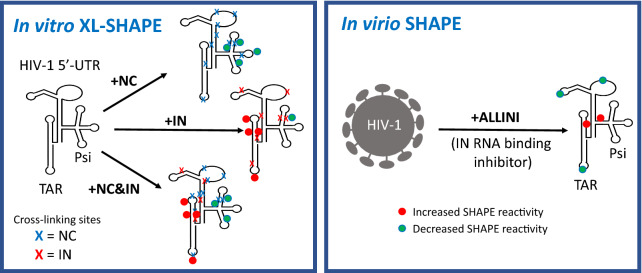

**Supplementary Information:**

The online version contains supplementary material available at 10.1186/s12977-021-00582-0.

## Background

Retroviruses such as HIV-1 undergo dramatic morphological changes during viral assembly and maturation that are essential to form mature infectious virions [1, 2]. Understanding these processes at the molecular level may lead to new therapies interfering with these critical steps. In the first step of viral assembly, Gag and Gag-Pol polyproteins assemble in a radial manner to form an immature particle. In the second step, immature HIV-1 particles undergo maturation wherein the polyproteins are cleaved by the viral protease and rearrange to form the infectious viral particle [3]. During this step, liberated capsid (CA) proteins assemble to form the mature capsid core, enclosing a viral ribonucleoprotein complex (vRNP) containing two copies of genomic RNA (gRNA) that are coated and condensed by nucleocapsid (NC) proteins; the vRNP also contains reverse transcriptase (RT) and integrase (IN) [1, 3]. The canonical role of HIV-1 IN is to catalyze insertion of the reverse transcribed viral DNA into host chromatin [4]. However, IN mutations have been identified that not only impair viral integration, but also influence other steps of the viral lifecycle, including maturation (reviewed in [5, 6]). Mutations that specifically impair viral DNA integration are known as class I, whereas class II mutations also affect other steps of the viral lifecycle [5]. Many class II IN mutations lead to aberrant virion morphogenesis and mis-localization of the vRNP outside the capsid core, resulting in non-infectious virions [5, 7–13]. Similarly, mutants with insertion of stop codons in the IN-coding region display abnormal virion morphogenesis [7, 14]. Supplying IN in *trans* to class II mutant virions partially restored vRNP encapsidation and HIV-1 infectivity, suggesting an active role for IN in HIV-1 particle morphogenesis [11].

Allosteric IN inhibitors (ALLINIs, also known as NCINIs, LEDGINs, or INLAIs) are a promising class of anti-retroviral drugs [15–20]. ALLINIs interact with the dimer interface of the IN catalytic core domain and induce aberrant IN multimerization [21–26]. The phenotype of virions produced from ALLINI-treated cells is very similar to that of class II IN mutations, i.e., mis-localization of vRNPs [11, 12, 15, 23, 24, 27, 28]. The mechanism of the ALLINI-induced effect was shown to be through disruption of IN-gRNA interactions [12]. Consistently, class II IN mutant viruses displayed impaired IN-gRNA interactions, although through different mechanisms including protein stability, which greatly reduced the packaged IN levels [13]. While IN-gRNA interactions are largely abolished by ALLINIs, mis-localized vRNPs still maintain a high NC content as determined by “tomo-bubblegram” imaging [11]. CLIP-seq studies also indicated that NC-gRNA interactions are unaltered in the presence of ALLINI treatment [12, 29]. The decrease in viral infectivity under ALLINI treatment is due to the premature loss of viral gRNAs and IN in target cells [29, 30].

Disruption of proper CA core formation around the vRNP appears to be a particularly attractive drug target [1, 3]. However, a detailed understanding of the interplay between key players in this process (NC, IN and gRNA) at the molecular level is lacking. A crosslinking study performed in cells revealed that NC and IN bind to gRNA in a largely orthogonal manner [12]. The HIV-1 gRNA 5′-untranslated region (5′-UTR) consists of multiple structural elements: the transactivation response element [TAR, nucleotide (nt) 1–58] and polyadenylation signal (polyA, nt 59–105) hairpins, the primer binding site domain (PBS, nt 126–224), and the packaging signal (Psi, nt 229–335) [31]. This region of the genome regulates diverse aspects of the viral life cycle, including gRNA dimerization, packaging, initiation of reverse transcription, RNA transcription and translation [32]. HIV-1 NC is known to specifically bind to Psi and mediate gRNA packaging [33–39]. A high-affinity IN binding site was identified in TAR, based on CLIP-seq and in vitro binding studies [12]; however, the interactions between IN and gRNA have not been probed at the single-nt resolution level. Therefore, in this work we focused on a detailed investigation of interactions of NC and IN with the gRNA 5′-UTR domain.

We applied crosslinking-coupled selective 2′-hydroxyl acylation analyzed by primer extension (XL-SHAPE), a single-nt resolution technique that allows the identification of both direct protein interaction sites and protein binding-induced RNA conformational changes [40]. Changes in SHAPE reactivity indicate altered flexibility of the RNA backbone, reflecting RNA conformational changes as a result of protein binding. Increased reactivity is likely due to an indirect effect, whereas decreased SHAPE reactivity may be due to indirect effects or direct protein binding. The crosslinking method is used to confirm direct sites of protein interaction. We probed the interactions of the HIV-1 5′-UTR, with IN or NC alone, and with both proteins simultaneously. Combined with results of SHAPE experiments performed in native virions both in the absence and presence of ALLINI treatment, our data provide significant new insights into IN/NC-gRNA 5′-UTR interactions in vitro and in virio.

## Results

### RNA and protein constructs, design of in vitro probing experiments, and data presentation

We first used SHAPE to probe the 352-nt HIV-1 5′-UTR RNA in the absence of added protein factors to ensure the RNA is properly folded. To avoid conformational heterogeneity due to monomer-dimer equilibrium, a monomeric mutant 5′-UTR-ΔDIS construct was used for all in vitro probing experiments, wherein the palindromic dimerization initiation signal (DIS) loop (AAGCGCGCA) was mutated to a stable GAGA tetraloop [41]. The sequence at the 5′-end of the HIV-1 genome has been shown to be heterogeneous, containing variable numbers of guanosines [42, 43]. RNAs with a single 5′ G are preferentially packaged, in part due to adopting a DIS-exposed conformation and having a higher propensity to dimerize [43, 44]. The conformation of the 5′-UTR-ΔDIS construct was largely insensitive to the number of 5′ Gs (data not shown) and a construct containing three 5′ Gs was used here. The lowest-energy secondary structure was obtained using RNAstructure [45], with the SHAPE data as pseudo-energy constraints. The secondary structure of the RNA (Additional file 1: Fig. S1) is consistent with previous in vitro probing results [46] and is also similar to the previously determined in virio 5′-UTR structure [38]. Therefore, the RNA conformation under our in vitro probing conditions resembles the authentic RNA conformation inside HIV-1 virions.

We present probing data for the following protein binding conditions: NC alone, IN/IBD alone and the two proteins together with different order of addition. IN/IBD refers to a complex between the IN-binding domain (IBD) of lens-epithelium-derived-growth-factor (LEDGF)/p75 and an F185H mutant form of HIV-1 IN. The IBD helps improve the solubility of IN and stabilizes the tetrameric form of IN that displays high binding affinity to RNAs [13, 47–49]. The F185H IN variant was used because this single point mutation increases the solubility of recombinant IN but does not affect viral replication [50]. In experiments wherein both proteins were present, NC or IN/IBD was added first, followed by titration of the second protein. To specifically assess the effect of the second added protein under these conditions, XL-SHAPE data of RNA complexes with the first protein were used as the background.

**Fig. 1:**
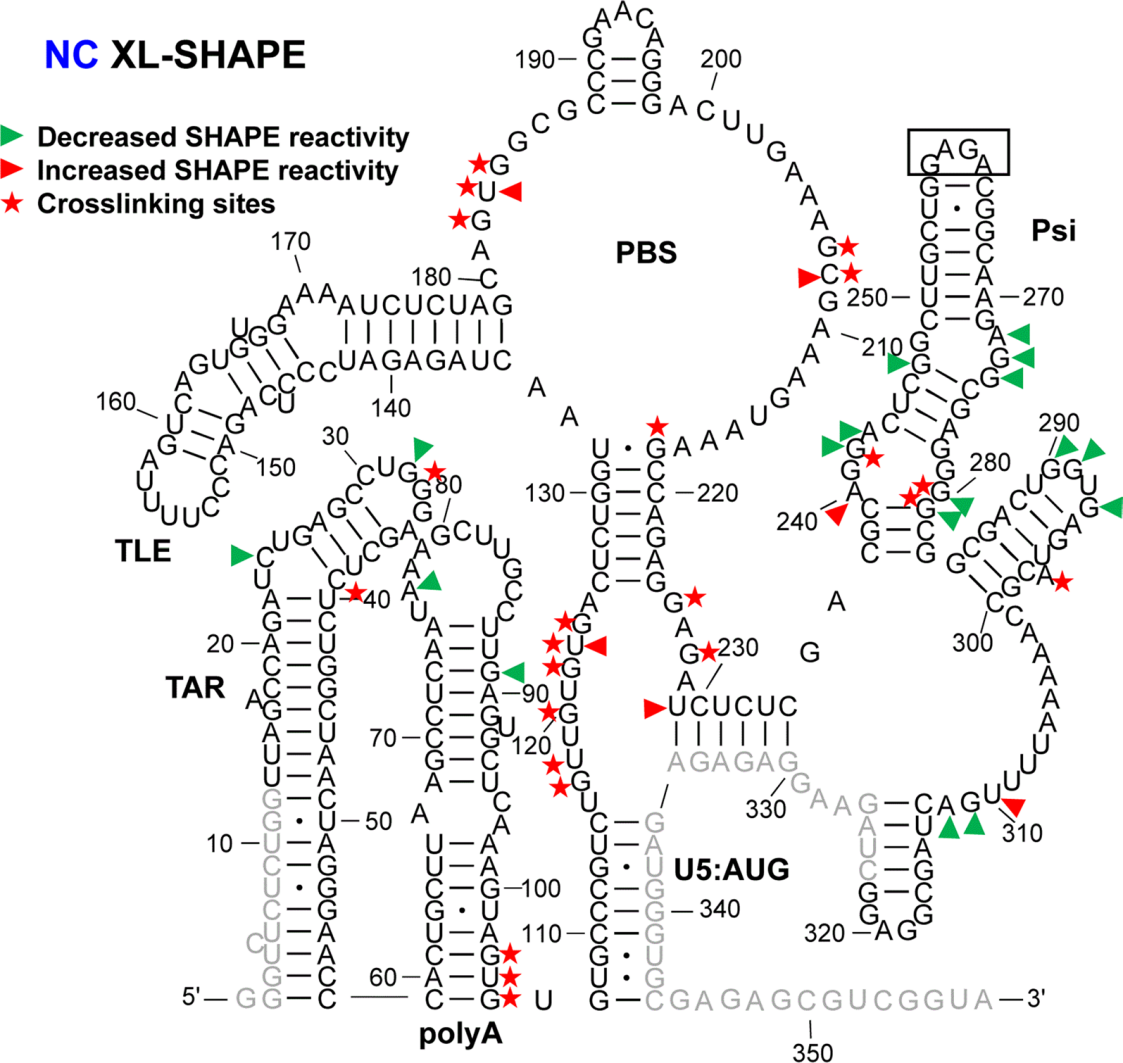
XL-SHAPE analysis of 5′-UTR RNA in presence of HIV-1 NC. The identified crosslinking sites are labeled by red stars. Sites with decreased and increased SHAPE reactivity upon protein binding are indicated by green and red arrowheads, respectively. All identified sites had reactivity changes of ≥ 0.3 and p < 0.05 based on unpaired, two-tailed Student’s t-tests, compared with the no protein control. Results are based on the average of at least 3 independent experiments. Nucleotides that could not be analyzed are shown in grey. In this construct, the Psi DIS sequence (AAGCGCGCA) was replaced by a GAGA tetraloop (boxed). Nucleotide numbering is according to the WT HIV-1 5′-UTR sequence

**Fig. 2:**
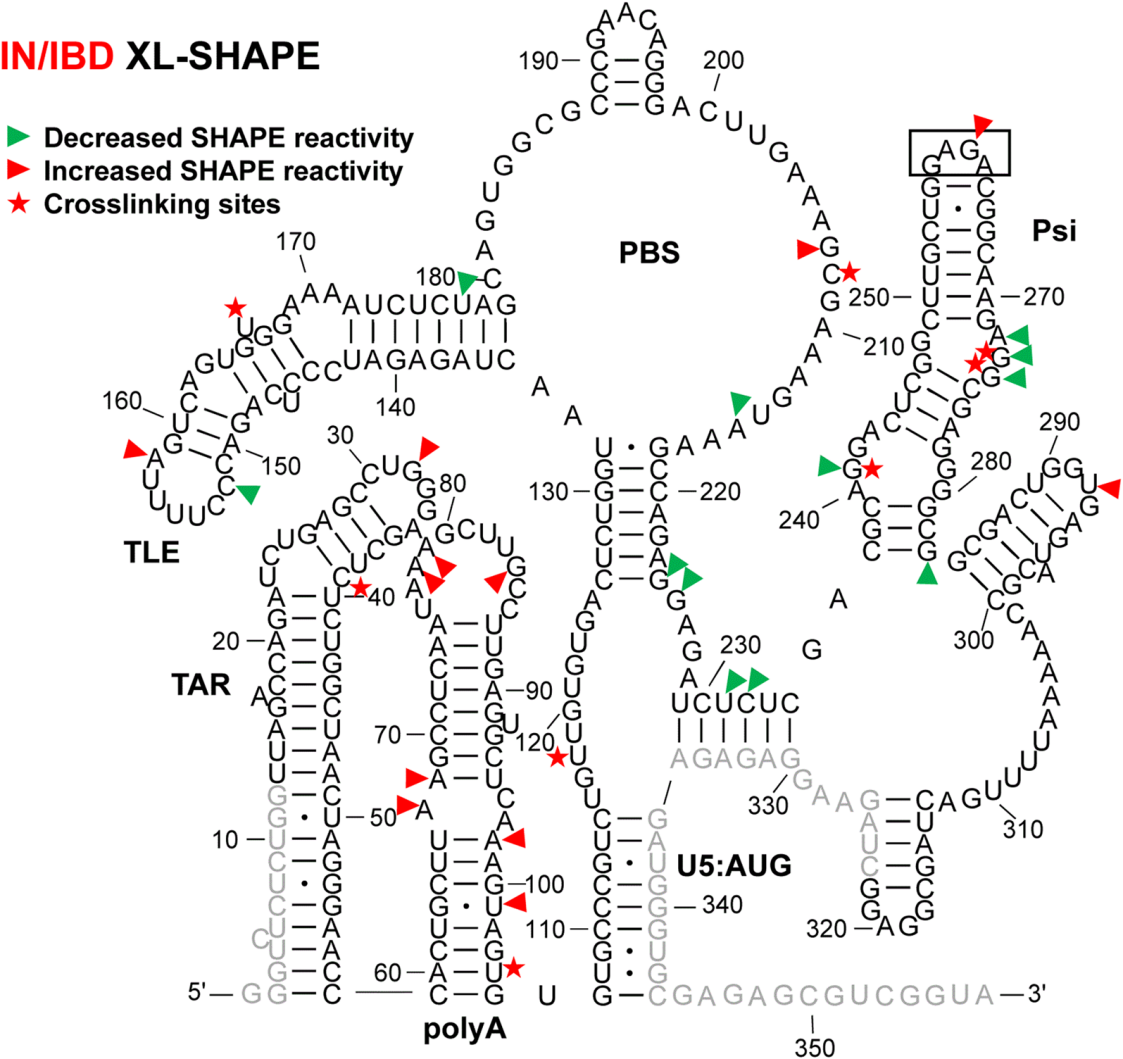
XL-SHAPE analysis of 5′-UTR RNA in presence of IN/IBD. The identified crosslinking sites are labeled by red stars. Sites with decreased and increased SHAPE reactivity upon protein binding are indicated by green and red arrowheads, respectively. All identified sites had reactivity changes of ≥ 0.3 and p < 0.05 based on unpaired, two-tailed Student’s t-tests, compared with the no protein control. Results are based on the average of at least 3 independent experiments. Other information is as noted in the legend to Fig. 1

**Fig. 3:**
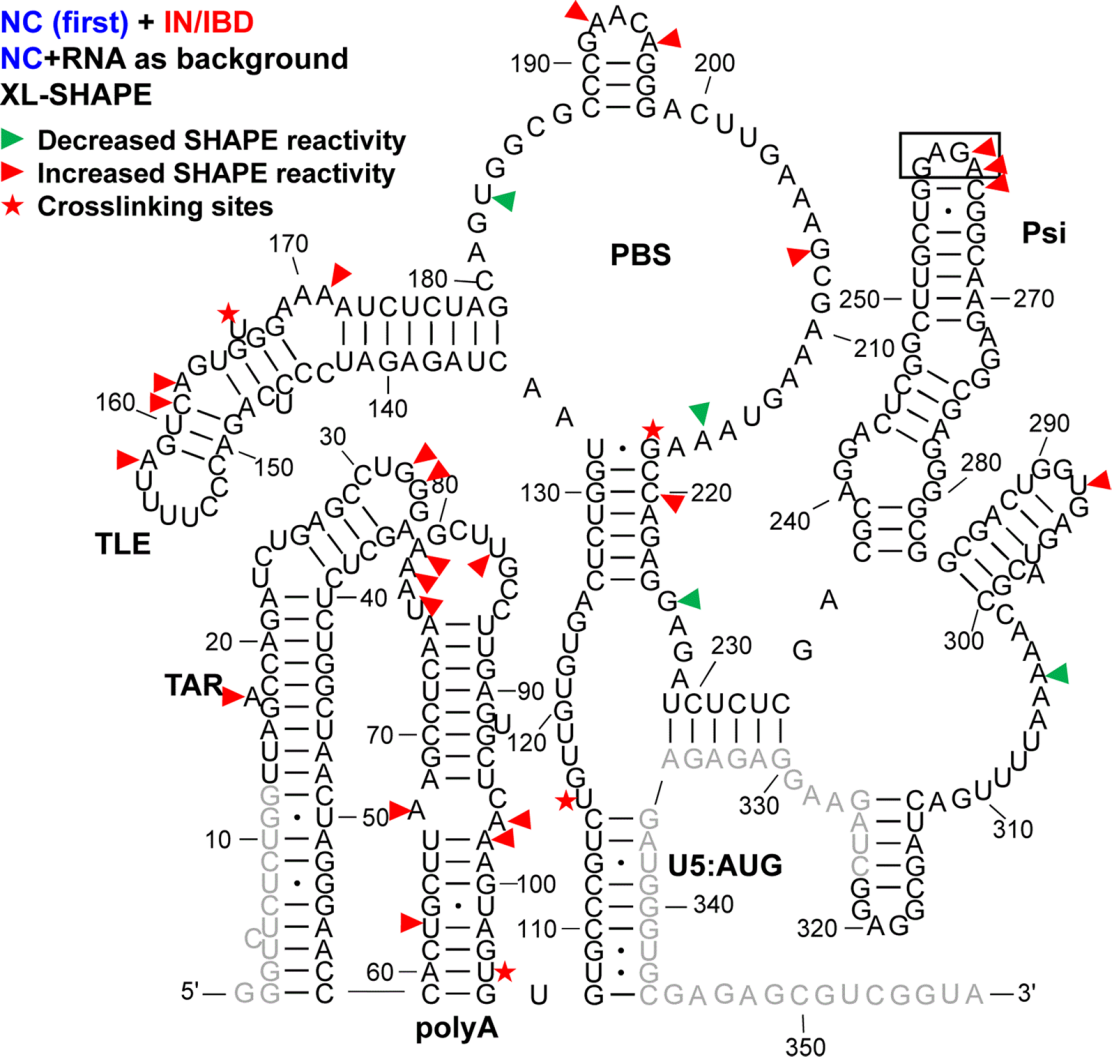
XL-SHAPE analysis of IN/IBD binding to the HIV-1 5′-UTR-ΔDIS/NC complex. The identified crosslinking sites are labeled by red stars. Sites with decreased and increased SHAPE reactivity upon protein binding are indicated by green and red arrowheads, respectively. All identified sites had reactivity changes of ≥ 0.3 and p < 0.05 based on unpaired, two-tailed Student’s t-tests, compared with the RNA + NC control. Results are based on the average of at least 3 independent experiments. Other information is as noted in the legend to Fig. 1

**Fig. 4:**
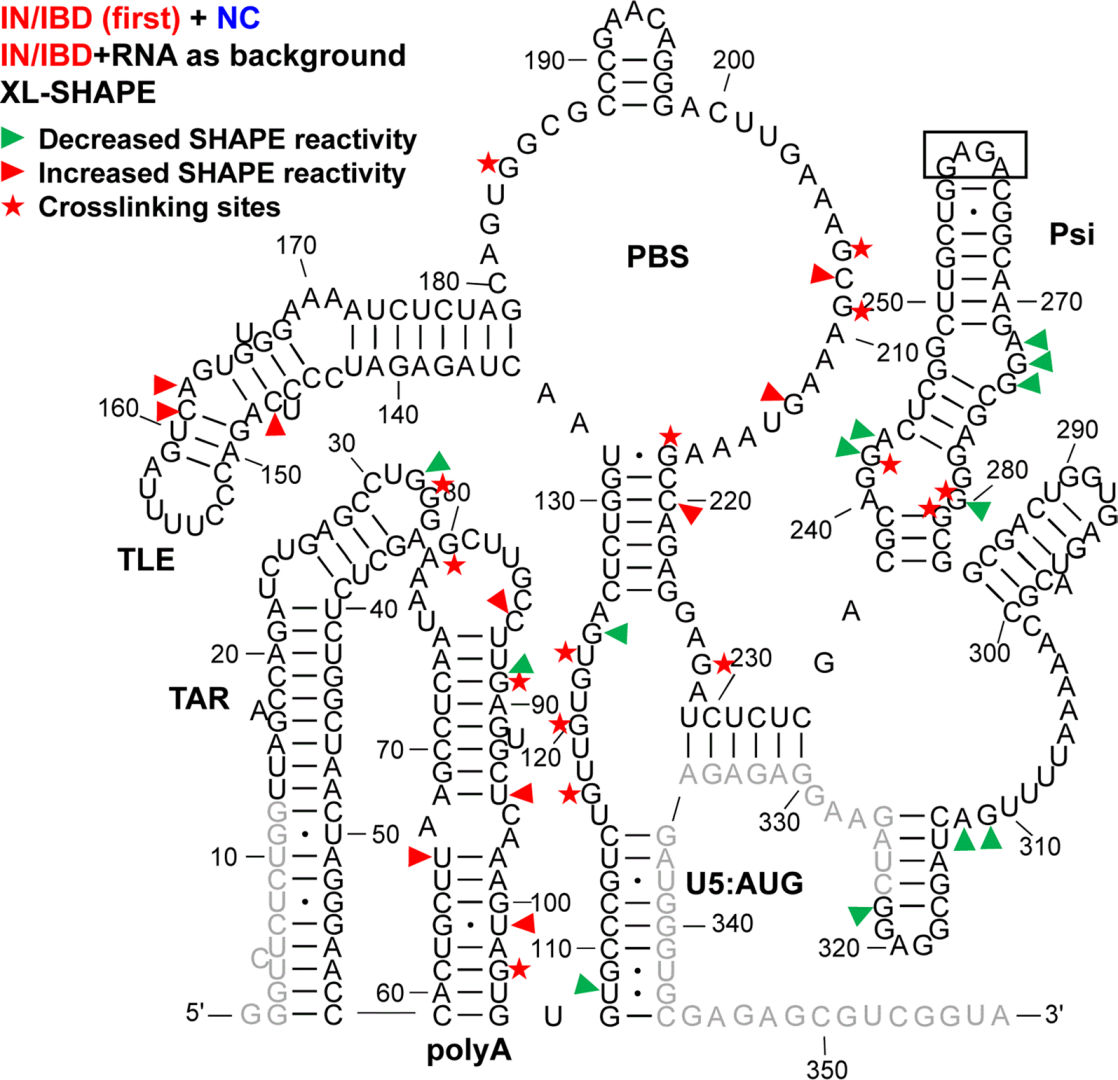
XL-SHAPE analysis of NC binding to the HIV-1 5′-UTR-ΔDIS/IN/IBD complex. The identified crosslinking sites are labeled by red stars. Sites with decreased and increased SHAPE reactivity upon protein binding are indicated by green and red arrowheads, respectively. All identified sites had reactivity changes of ≥ 0.3 and p < 0.05 based on unpaired, two-tailed Student’s t-tests, compared with the RNA + IN/IBD control. Results are based on the average of at least 3 independent experiments. Other information is as noted in the legend to Fig. 1

A summary of the results for the 5′-UTR under each protein binding condition is presented in Figs. 1, 2, 3 and 4. To facilitate comparisons of the effect of the different proteins, data obtained with each protein combination are summarized separately for each of the major 5′-UTR domains (Additional file 2: Fig. S2, Additional file 3: Fig. S3, Additional file 4: Fig. S4, and Additional file 5: Fig. S5). The statistical significance of the identified crosslinking sites and SHAPE reactivity changes were assessed by the two-tailed Student’s t-test, and the indicated changes were all dose-dependent.

### IN/IBD binding results in increased SHAPE reactivity of the apical TAR loop and polyA hairpin independent of NC binding

TAR plays a major role in stimulating viral transcription [51], whereas the polyA hairpin has been shown to regulate gRNA production and packaging [36]. These hairpins together adopt a stable coaxially stacked conformation [41]. NC alone crosslinked to sites in both TAR and polyA hairpins (Fig. 1). As indicated by the sites with decreased SHAPE reactivity (green arrowheads), NC binding led to decreased backbone flexibility in the internal and apical loops of TAR and there were two significant sites with decreased SHAPE reactivity in polyA. In the case of IN/IBD alone, we observed one significant crosslinking site at C39 in TAR (Fig. 2). This is consistent with a previous CLIP-seq study showing that the TAR loop is an IN interaction site in virions [12]. IN/IBD was also observed to crosslink at the 3′-end of the polyA hairpin. Although two of the observed crosslinking sites for NC and IN/IBD overlapped (Figs. 1 and 2), IN/IBD binding largely increased SHAPE reactivity in the apical loop of TAR and the polyA hairpin, in contrast to the decrease reactivity observed upon NC binding (Fig. 2). Although most of the sites with significantly increased SHAPE reactivity were in or near the single-stranded regions of polyA, there was an overall trend of increased SHAPE reactivity across the entire polyA hairpin (Additional file 10).

When NC was preincubated with the 5′-UTR before IN/IBD addition, the C39 crosslinking site observed for IN/IBD alone was lost (Fig. 3); however, IN/IBD binding still resulted in increased SHAPE reactivity in the TAR apical loop and the polyA hairpin (Fig. 3), suggesting that increased nt flexibility upon IN/IBD binding was unaffected by the presence of NC. When IN/IBD was preincubated with the 5′-UTR followed by NC addition, a similar although not identical pattern of XL-SHAPE reactivity was observed as with NC alone (Fig. 4). The preincubation with IN/IBD led to new NC crosslinking sites (G89, G80) in polyA, possibly because IN/IBD binding destabilized the backbone, making these sites more accessible. G89 also showed significantly lower SHAPE reactivity, consistent with the crosslinking result. In contrast to the NC only results, preincubation with IN/IBD triggered significantly increased SHAPE reactivity in multiple polyA sites upon NC binding (Fig. 4). The changes in TAR/polyA are summarized in Additional file 2: Fig. S2.

### NC shows strong crosslinking to a UG-rich single-stranded region in U5

Upon binding of NC alone, numerous crosslinking sites were observed in the single-stranded region of U5 (Fig. 1). This region is highly UG-rich, which is consistent with NC’s preferred binding motif [39, 52, 53]. Two additional crosslinking sites were observed in the single-stranded region that connects the PBS and Psi domains. These sites are consistent with the previously identified NC interaction sites from in virio SHAPE and in vitro binding studies [38, 54]. For IN/IBD alone, one significant crosslinking site (U118) was identified in the single-stranded region of U5 (Fig. 2). Decreased SHAPE reactivity was observed in the single-stranded region between PBS and Psi (Fig. 2). In the presence of preincubated NC, IN/IBD still crosslinked to the single-stranded region near U5:AUG (Fig. 3). However, the presence of NC largely prevented the IN/IBD-induced decrease in SHAPE reactivity of the single-stranded region between PBS and Psi (Fig. 3). When IN/IBD was pre-bound, NC still crosslinked to this region of the RNA (Fig. 4). The changes in the U5:AUG region are summarized in Additional file 3: Fig. S3.

### Variable SHAPE reactivity changes of the PBS/TLE domain

The PBS domain contains sequences complementary to the 3′-end 18 nt of human tRNA^Lys,3^, the HIV-1 reverse transcription primer, and is important for reverse transcription initiation [55]. Within the PBS domain, there is also a hairpin known as the tRNA-like element (TLE, nt 149–161), which mimics the tRNA^Lys,3^ anticodon domain [41, 56, 57]. Several crosslinking sites were observed for NC alone that were consistent with this protein’s preference for single-stranded guanosines and UG motifs (Fig. 1). Three crosslinking sites (G182, U183 and G184) are proximal to where the 3′-end of the tRNA^Lys,3^ primer anneals. NC binding also increased the SHAPE reactivity at these crosslinking sites, indicating that NC binding may make the PBS region more accessible to the tRNA^Lys,3^ primer. IN/IBD alone crosslinked to U166 near the TLE hairpin and C208, downstream from the PBS, resulting in mixed decreased and increased SHAPE reactivity (Fig. 2). When NC was preincubated with the 5′-UTR, IN/IBD still crosslinked to U166 (Fig. 3). A second crosslinking site was retained under these conditions, though shifted from C208 to G218, while maintaining mixed decreased and increased SHAPE reactivity. When IN/IBD was preincubated with the RNA, a crosslinking pattern reminiscent of NC alone was observed (Fig. 4). New SHAPE reactivity changes, however, were observed near the TLE hairpin, suggesting that NC triggered increased nt flexibility under these conditions. In the single-stranded region of the PBS domain, several sites with increased SHAPE reactivity changes were also observed (Fig. 4). The changes in the PBS/TLE domain are summarized in Additional file 4: Fig. S4.

### NC bound Psi independent of IN/IBD binding

The Psi domain, which is responsible for directing gRNA packaging via interactions with the NC domain of the Gag polyprotein [58–60] (also reviewed in [61–63]), is composed of three stem-loops: SL1, SL2 and SL3. As expected based on the known binding specificity of NC to Psi [33–37], NC crosslinked to a G-rich bulge region near the base of the SL1 hairpin, which correlated with decreased SHAPE reactivity changes (Fig. 1). Significantly decreased SHAPE reactivity changes were also observed in the upper single-stranded bulge region of SL1, as well as in a single-stranded region near the base of SL3. Although the decreased shape reactivity was consistent with NC binding to these regions, significant levels of crosslinking were not observed in the vicinity of the SL3 base. The decreased SHAPE reactivity in the G-rich apical loop of SL2 was also consistent with previous in virio SHAPE probing results [38]. IN/IBD also crosslinked to the single-stranded regions in SL1, consistent with the decrease in SHAPE reactivity it invoked in these regions (Fig. 2). In contrast to NC, IN/IBD led to increased SHAPE reactivity in the apical loop regions of both SL1 and SL2. When NC was preincubated with the RNA, IN/IBD crosslinking to the Psi domain was abolished and SHAPE reactivity changes in SL1 were also largely eliminated (Fig. 3). Interestingly, the converse was not observed. That is, upon preincubation with IN/IBD, NC crosslinking sites and NC binding-induced SHAPE reactivity changes in SL1 were very similar to the results obtained with NC alone (Fig. 4). The changes in Psi are summarized in Additional file 5: Fig. S5.

### IN-specific XL-SHAPE reactivity effects in vitro

To assess potential contributions from the IBD protein to the XL-SHAPE changes observed upon IN/IBD binding, we carried out XL-SHAPE analysis with IBD only (Additional file 6: Fig. S6). The IBD did not reveal any crosslinking sites, indicating it does not stably interact with RNA. Although SHAPE reactivity changes were observed for the IBD, these affected only 11 RNA sites compared to 22 sites with the IN/IBD complex (Fig. 2). Also, in the vast majority of cases, the sites affected by the IBD alone differed from the IN/IBD complex (compare Fig. 2 and Additional file 6: Fig. S6). The only alteration in common to both the IBD and IN/IBD complex was G224 reactivity. Collectively, these results indicate that the IBD does not significantly contribute to the RNA binding profile of the IN/IBD complex.

**Fig. 5:**
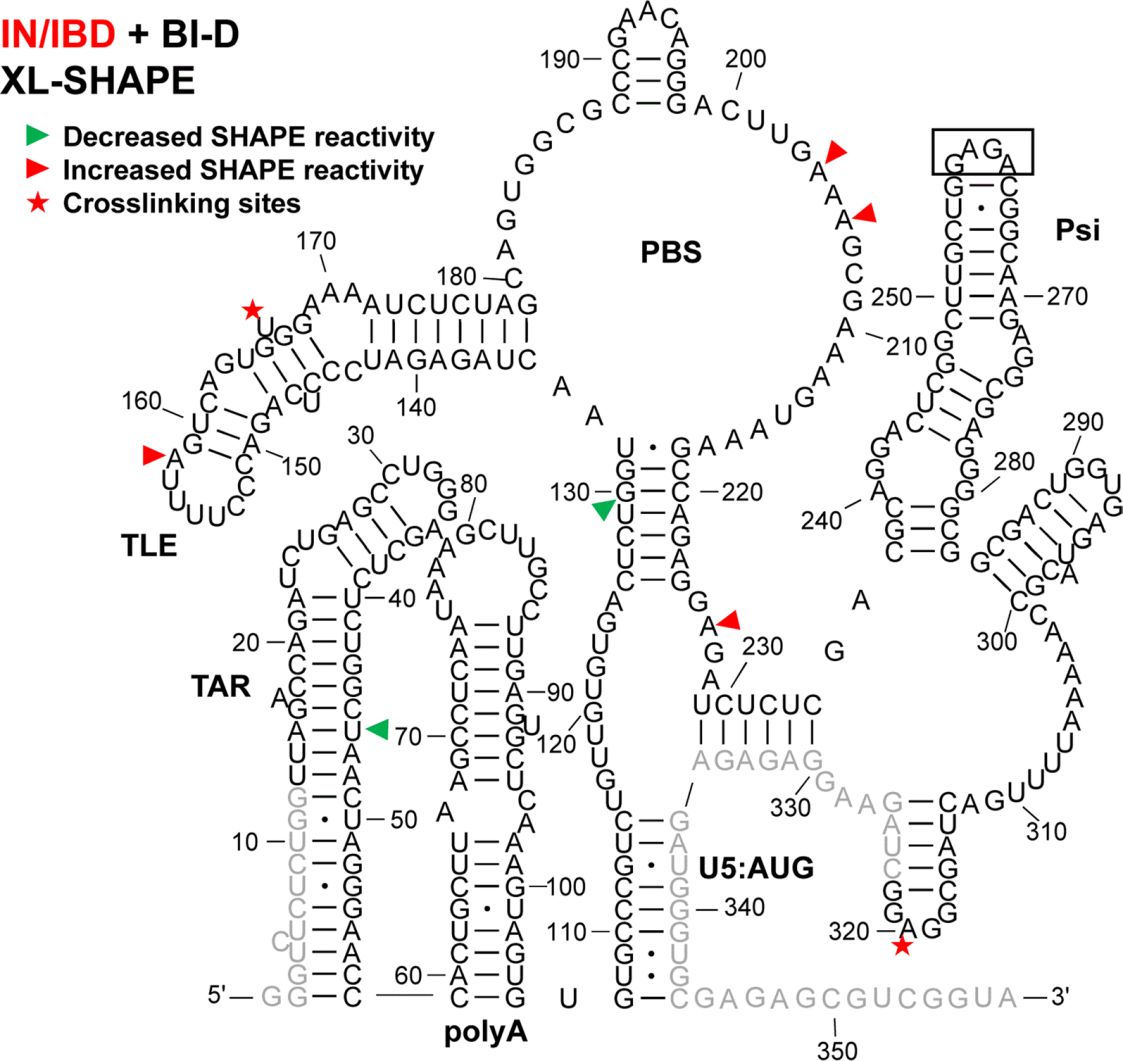
XL-SHAPE analysis of IN/IBD complexes that had been pretreated with ALLINI BI-D. The identified crosslinking sites are labeled by red stars. Sites with decreased and increased SHAPE reactivity upon protein binding are indicated by green and red arrowheads, respectively. All identified sites had reactivity changes of ≥ 0.3 and p < 0.05 based on unpaired, two-tailed Student’s t-tests, compared with the no protein control. Results are based on the average of at least 3 independent experiments. Nucleotides that could not be analyzed are shown in grey. In this construct, the Psi DIS sequence (AAGCGCGCA) was replaced by a GAGA tetraloop (boxed). Nucleotide numbering is according to the WT HIV-1 5′-UTR sequence

An orthologous control experiment was performed with the IN/IBD pretreated with BI-D, a well-characterized quinoline ALLINI [11, 12]. It was previously shown that exposure of HIV-1 in cells to ALLINIs during virus egress impaired IN-RNA binding in virions [12]. The XL-SHAPE results obtained with the BI-D-treated IN/IBD-RNA complex were mapped onto the RNA secondary structure (Fig. 5). Compared to the results obtained with untreated IN/IBD complexes (Fig. 2), BI-D pretreatment significantly reduced XL-SHAPE effects. For example, BI-D-treated IN/IBD failed to increase SHAPE reactivity in the TAR apical loop and polyA hairpin and failed to decrease SHAPE reactivity in Psi. The five sites of IN crosslinking in these regions observed under baseline conditions were also absent. In contrast, one crosslinking site (U166) in the TLE hairpin and one site with significantly increased SHAPE reactivity in the TLE loop were retained following BI-D pretreatment. Taken together, these data support specific IN-RNA binding in the TAR/polyA and Psi regions of the 5′-UTR, which were adversely affected by ALLINIs.

As an additional control, an IN mutant (R269A, K273A) with impaired RNA binding [12] was tested. The XL-SHAPE results are shown in Additional file 7: Fig. S7. As expected, we no longer observed any significant crosslinking sites, consistent with an RNA-binding defect. This IN mutant also induced far fewer significant SHAPE reactivity changes, compared to WT IN/IBD, especially in TAR/polyA. There were still several sites with significant SHAPE reactivity changes in Psi, suggesting that these may be less specific effects.

### In vitro probing of 5′-UTR-protein complexes with near-physiological NC stoichiometry

HIV-1 virions incorporate two copies of gRNA (~ 9.4 kilobases each), ~ 2500 copies of NC, and ~ 125 copies of IN [64, 65]. Therefore, the stoichiometry of NC:nt and IN:nt is about 1:8 and 1:160, respectively. In the XL-SHAPE experiments described thus far, the IN and RNA concentrations that were used matched physiological stoichiometries. However, a lower NC concentration (up to a maximum of 1NC: 30 nt) was used to maintain dose-dependent reactivity changes; when higher concentrations were used, dose dependence was no longer observed, potentially due to NC saturation or NC-induced RNA aggregation. We next compared the results obtained at lower NC concentrations to experiments using near-physiological NC/RNA stoichiometries. The result of an XL-SHAPE experiment wherein 5′-UTR-protein complexes were probed following IN/IBD pre-incubation and addition of NC at 1NC:8nt is shown in Additional file 8: Fig. S8. As before, the reactivity of the IN/IBD-RNA complex was used as the background to assess the reactivity changes induced by NC addition. Overall, the results were very similar to those observed previously at lower NC concentration (Fig. 4), with the exception of additional sites of increased SHAPE reactivity in the PBS/TLE domain.

### In virio probing following ALLINI treatment leads to specific local SHAPE reactivity changes in the 5′-UTR

**Fig. 6:**
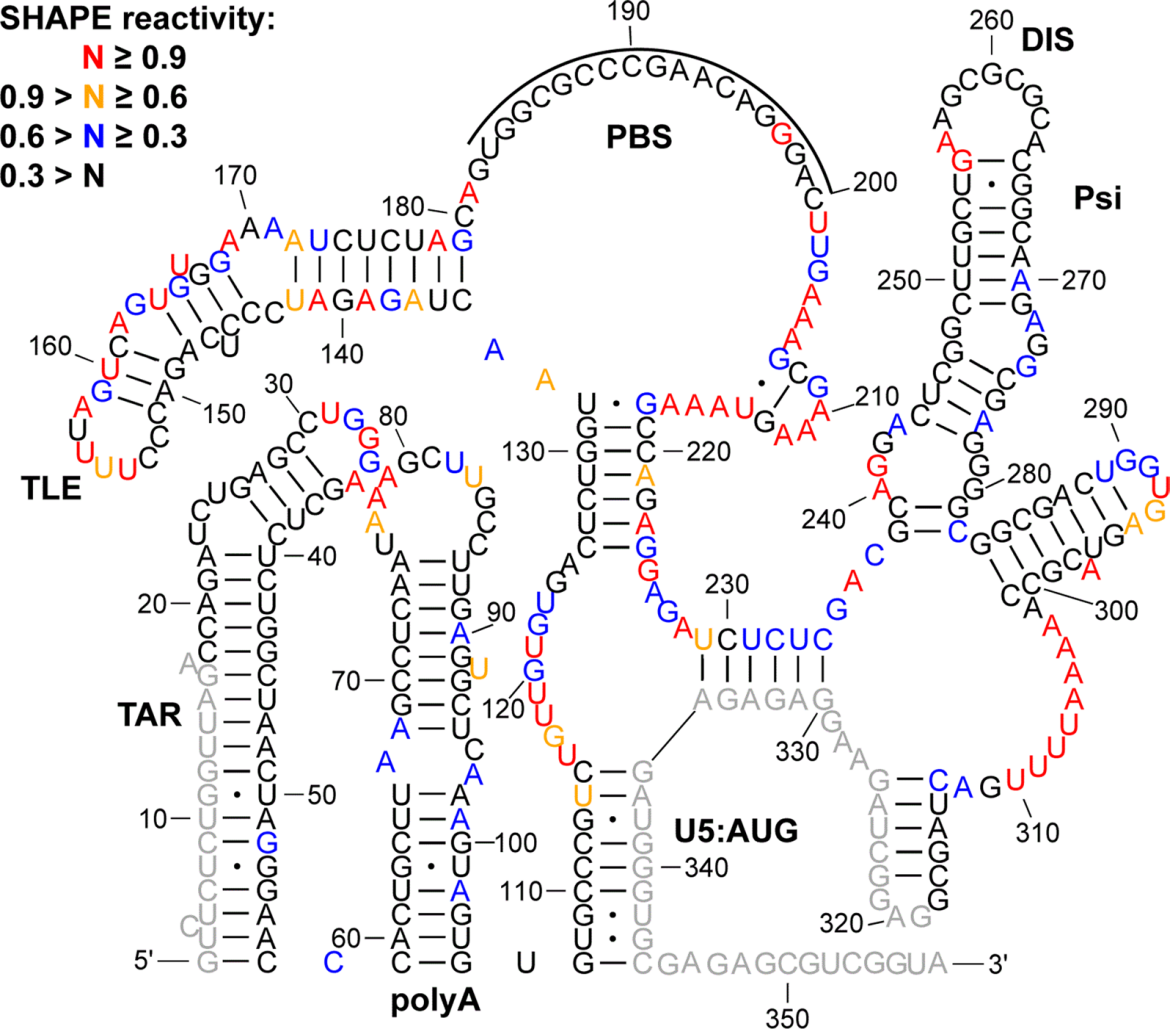
In virio SHAPE reactivity-constrained lowest energy secondary structure of the HIV-1 5′-UTR, after treatment of cells with 10 µM BI-D. The secondary structure model was generated by applying averaged normalized SHAPE reactivity from two independent trials as pseudo free-energy constraints. Nucleotides are colored according to SHAPE reactivity as indicated in the key. Nucleotides that could not be analyzed are shown in grey. The tRNA^Lys,3^ annealing site is indicated by a black line

Next, we performed in virio SHAPE probing of RNA extracted from virions to assess how inhibition of IN-RNA binding by ALLINI treatment affects global and local 5′-UTR structures. To abolish IN-RNA binding, viral producer cells were exposed to BI-D during HIV-1 egress [12]. In the absence of BI-D treatment, the SHAPE reactivity-constrained lowest energy secondary structure of the 5′-UTR (Additional file 9: Fig. S9) was almost identical to the previous in virio SHAPE structure [38]. Likewise, when the producer cells were treated with BI-D, the lowest energy secondary structure was the same as the structure obtained in the dimethyl sulfoxide (DMSO) control experiments (Fig. 6), suggesting that BI-D treatment did not impart global secondary structure changes throughout the 5′-UTR region probed in our assay. We also derived lowest energy secondary structures of the 5′-UTR using in vitro SHAPE data with bound protein factors, and none of these structures differed from the structure of RNA alone (Additional file 1: Fig. S1). The fact that the derived structures with bound proteins were identical to the structure of RNA alone indicates that protein binding did not significantly affect global 5′-UTR secondary structure.

**Fig. 7:**
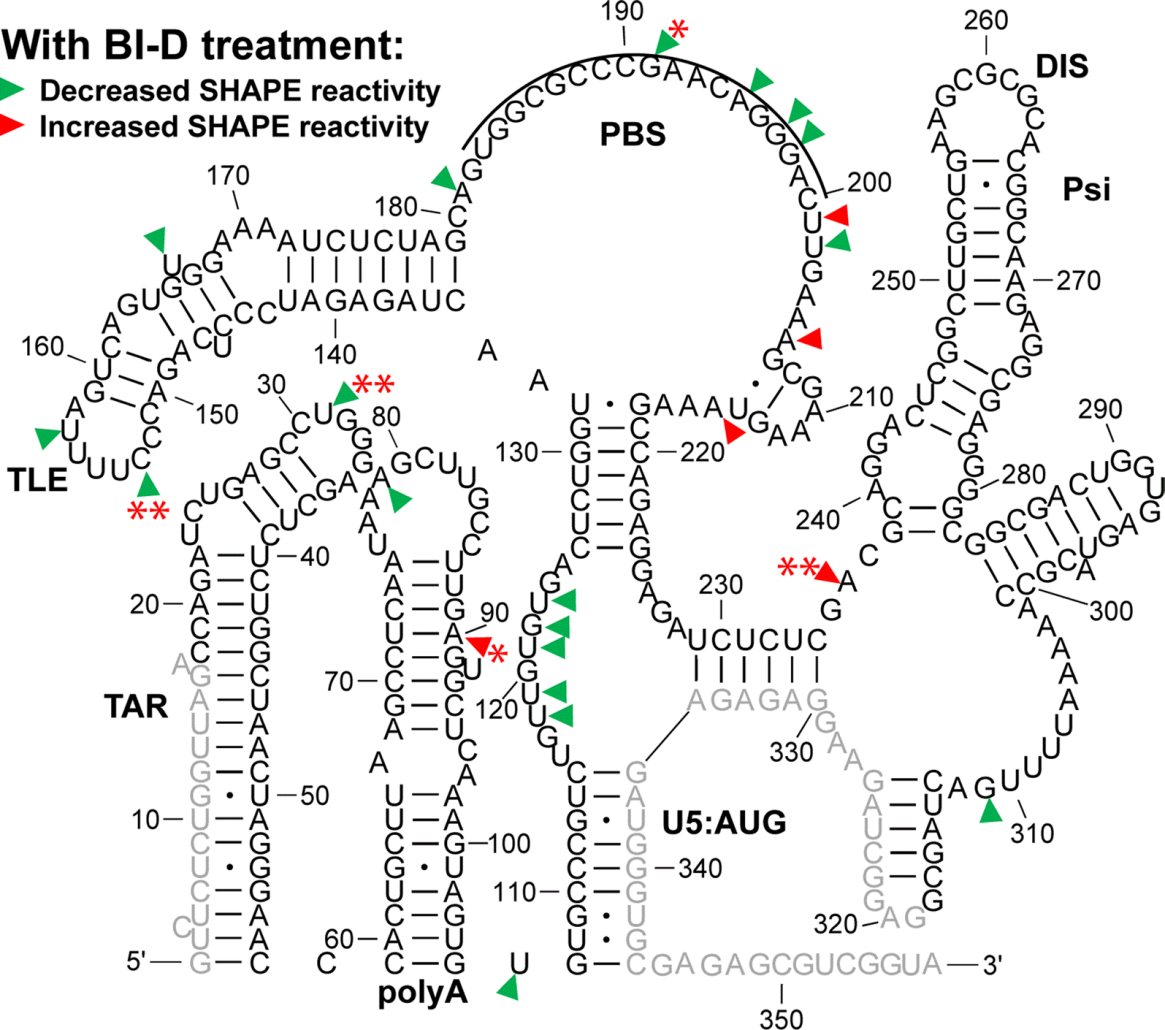
In virio SHAPE reactivity differences of the HIV-1 5′-UTR upon 10 µM BI-D treatment, as compared to DMSO-treated controls. Sites with decreased and increased SHAPE reactivity upon BI-D treatment are indicated by green and red arrowheads, respectively. All identified sites had reactivity changes of ≥ 0.3. Results are based on the average of 2 independent experiments. Nucleotides that could not be analyzed are shown in grey. Asterisks represent the results of two-tailed, unpaired Student’s t-test: (*): 0.05 ≤ p < 0.1; (**): p < 0.05). The tRNA^Lys,3^ annealing site is indicated by a black line

Upon BI-D treatment, some local SHAPE reactivity changes were, however, observed. Decreased SHAPE reactivity was detected throughout the 5′-UTR, with the most significant changes occurring in the TLE and TAR apical loops, as well as the PBS (Fig. 7). The most significant effects were observed at U31 (TAR loop, decrease), A90 (polyA stem, increase), C153 (TLE loop, decrease), G191 (PBS, decrease), and A236 (Psi, increase). This suggests that inhibiting IN-RNA binding alters nt flexibility in these regions. The decreased in virio SHAPE reactivity upon BI-D treatment in the apical TAR loop is consistent with the increased in vitro SHAPE reactivity upon IN/IBD binding (Figs. 2 and 3). As mentioned above, the TAR loop was identified as a high-affinity IN binding site based on CLIP-seq and in vitro binding studies [12]. While many nt in the polyA hairpin showed increased SHAPE reactivity upon IN/IBD binding in the in vitro XL-SHAPE experiments performed in the absence or presence of NC (see Figs. 2 and 3), we did not observe a corresponding significant decrease in SHAPE reactivity at the majority of these sites upon BI-D treatment of virions (Fig. 7). This may be due to the presence of other factors in virions that are missing in our in vitro system.

## Discussion

A previous CLIP-seq study of RNA extracted from HIV-1 virions suggested that U31 in the apical loop of TAR is a direct IN interaction site [12]. In our in vitro study, U31, G32 and G33 revealed significantly higher SHAPE reactivity, or increased flexibility, upon IN/IBD binding, with G32 displaying clear dose-dependent reactivity changes (Fig. 2 and Additional file 10). We observed increased crosslinking at C30 and U31 in the presence of IN/IBD (Additional file 10), though these changes were not designated as significant once we applied our stringent criteria. C39 in TAR was a significant site of IN crosslinking in our study. Significant increases in SHAPE reactivity were observed in the polyA domain upon IN/IBD binding and importantly, these increases were largely maintained even upon preincubation with NC. Interestingly, NC crosslinked to G33 in the TAR loop irrespective of IN/IBD binding. When IN/IBD was preincubated with the RNA, we observed several additional NC crosslinking sites (G80, G89) in polyA (Fig. 4). Overall, these data suggest that IN binding has a primary impact on nt flexibility in the polyA hairpin, with a more minor influence on TAR.

A previous in virio SHAPE study suggested that the ejection of Zn^2+^ from the NC zinc fingers, which results in defective NC-RNA binding, led to increased SHAPE reactivity changes in several single-stranded regions of Psi: two bulges in SL1 (nt 240–243, nt 272–274) and the apical loop of SL2 [38]. Our data are consistent with decreased SHAPE reactivity in these regions upon NC binding (Fig. 1). While many previous studies also suggested the importance of SL3 in NC binding and gRNA packaging [38, 54, 66, 67], we were unable to analyze this region for technical reasons related to overlap with the SHAPE primer annealing site. Within Psi, most of the sites with SHAPE reactivity changes and identified crosslinking sites were found in SL1, consistent with many previous studies suggesting SL1 is an important determinant for Gag recognition and gRNA packaging [33, 37, 68, 69]. We also observed several NC crosslinking sites and increased SHAPE reactivity changes near the PBS (Fig. 1), in good agreement with recent Rous sarcoma virus Gag crosslinking results [70].

IN and NC were previously demonstrated to bind to the gRNA in an orthogonal manner in virio [12]. Here, our SHAPE results suggest that IN/IBD binding triggered increased backbone flexibility of the apical loop of TAR and the polyA hairpin, regardless of the presence of NC, while NC preferentially bound to the single-stranded regions in U5, the region between PBS and Psi, and the Psi domain, regardless of the presence of IN/IBD. Therefore, the effect of NC and IN binding to the 5′-UTR is also orthogonal in vitro. We also observed some synergistic effects. For example, in the polyA hairpin, the presence of IN/IBD led to enhanced NC crosslinking (Figs. 1 and 4). In addition, the XL-SHAPE results suggested that IN/IBD interacts with the single-stranded bulges of SL1 only in the absence of NC (Figs. 2 and 3).

In virio SHAPE studies performed after treating producer cells with the ALLINI BI-D revealed that the lowest-energy secondary structure of the 5′-UTR was the same as the DMSO control (Fig. 6 and Additional file 9: Fig. S9). This was not surprising, as previous in virio SHAPE studies suggested that the secondary structure of HIV-1 gRNA is strongly conserved across different biological states (in vitro, ex virio, in virio) [38]. Additionally, ejection of Zn^2+^ from NC zinc fingers did not change the overall structure of the 5′-UTR in virio [38]. Therefore, our data and previous probing data [38] collectively suggest that IN and NC binding in vitro and in virio do not induce global secondary structural changes within the 5′-UTR. Our in virio SHAPE reactivity profiles in the PBS/TLE region (Additional file 9: Fig. S9) are also consistent with previous studies showing limited SHAPE reactivity at the PBS, and high SHAPE reactivity in the sequences downstream from the TLE loop due to additional tRNA/5′-UTR interactions [38, 71]. The DIS and the single-stranded bulges in SL1 were also unreactive, consistent with intermolecular interactions in the DIS and protection due to NC binding.

Our in vitro experiments suggested that NC specifically interacts with Psi, independent of the presence of IN. Treatment with ALLINIs was previously shown not to affect NC-RNA binding [29]. Consistent with this finding, our in virio SHAPE probing experiments suggested that pretreatment of virus with the ALLINI BI-D did not induce local SHAPE reactivity changes in Psi.

During viral maturation, the stability of the HIV-1 RNA dimer increases in a stepwise manner, and this process largely depends on the HIV-1 protease [72, 73]. The SHAPE reactivity of the viral RNAs may vary in virions of distinct ages. Future work aimed at probing viral RNAs from virions of different ages or protease-deficient virions may be beneficial to understand changes in RNA conformation during viral maturation.

## Conclusions

Our detailed investigation of in vitro binding of NC and IN with the HIV-1 5′-UTR is consistent with the conclusion that binding is largely orthogonal. NC binds preferentially to the UG-rich region proximal to the U5:AUG stem and Psi, while IN binding impacts nt flexibility in the TAR/polyA domains. The global secondary structure of the 5′-UTR remained unaltered in native vs. eccentric virions produced in the presence of ALLINIs. Instead, inhibition of IN-RNA interactions by ALLINIs changed local RNA backbone flexibility at a few specific sites throughout the 5′-UTR, including the apical TAR loop.

## Methods

### Preparation of proteins and RNAs

His_6_-tagged IN(F185H) and His_6_-tagged IBD (residues 307–460) [74, 75] were co-purified to form the IN (F185H)-IBD complex by using three chromatographic steps. The protocol to purify IN as described previously [76] was used with minor modifications. Briefly, both IN and IBD were expressed in BL21 (DE3) strain of *Escherichia coli* and the cells were grown at 37 °C in LB containing 120 µg/mL ampicillin for IN and 50 µg/mL kanamycin for IBD. The cells were grown to ~ 0.6–0.8 OD_600_, followed by 1 mM isopropyl β-d-1-thiogalactopyranoside induction for 4 h at 37 °C. Both IN and IBD cell pellets were lysed together in 50 mM 4-(2-hydroxyethyl)-1-piperazineethanesulfonic acid (HEPES), pH 7.5, 1 M NaCl, 10% glycerol, 2 mM 2-mercaptoethanol (BME) and protease inhibitor. After sonication, the filtered supernatant was subjected to nickel-affinity and heparin column purification as previously described [76]. This was followed by size-exclusion chromatography using a HiLoad 16/600 Superdex column (GE healthcare, Chicago, IL) in 50 mM HEPES, pH 7.5, 800 mM NaCl, 5% glycerol and 3 mM BME. The fractions were collected, concentrated and frozen at − 80 °C. A similar procedure was followed for purifying His_6_-tagged IBD alone. His_6_-tagged IN(R269A, K273A) was purified as described [12, 48].

HIV-1 NC protein (BH10 strain) was purchased as a synthetic peptide from New England Peptide (Gardner, MA).

HIV-1 352-nt 5′-UTR-ΔDIS (NL4-3 strain) was prepared by in vitro transcription using FokI-linearized pUC19 plasmid templates and T7 RNA polymerase, as described [77]. This RNA contains a GAGA tetraloop mutation in replacement of the DIS. The RNA concentration was determined by measuring the absorbance at 260 nm and the following molar extinction coefficient: 3.2 × 10^6^ M/cm.

### In vitro XL-SHAPE probing

Prior to all XL-SHAPE probing experiments, the HIV-1 5′-UTR-ΔDIS RNA (4 µM) was refolded in 50 mM HEPES, pH 7.5 by heating to 80 °C for 2 min, 60 °C for 2 min, addition of 1 mM MgCl_2_, followed by incubation at 37 °C for 30 min and incubation on ice for at least 30 min.

An initial SHAPE probing experiment was performed to test whether the RNA was properly folded in the absence of bound protein. For this experiment, the folded RNA was diluted to 0.1 µM in 20 mM HEPES, pH 7.5 and 1 mM MgCl_2_. Diluted RNA (90 µL) was mixed with either 10 µL of neat DMSO or 10 µL of *N*-methylisatoic anhydride (NMIA, from Sigma Aldrich, St. Louis, MO) (8 mM in DMSO). The SHAPE reactions were incubated at 37 °C for 45 min and the RNAs were recovered by ethanol precipitation.

For XL-SHAPE probing experiments with NC, IN/IBD, or IN(R269A, K273A), the folded RNAs were diluted to 0.1 µM and incubated with each protein for 2 h on ice, in a buffer containing 20 mM HEPES, pH 7.5, 1 mM MgCl_2_ and 100 mM NaCl. The following protein concentrations were used: 1, 0.7, 0.3 and 0 µM for NC; 0.3, 0.2, 0.1 and 0 µM for IN/IBD, and 0.3, 0.1, 0 µM for IN(R269A, K273A). For XL-SHAPE probing experiments with BI-D-treated IN/IBD, 10-fold molar excess of BI-D (in DMSO) was added to each diluted IN/IBD complex, followed by incubation at room temperature for 1 h. For XL-SHAPE experiments with both NC and IN/IBD, the folded RNA was diluted to 0.125 µM and first incubated with 1.25 µM NC or 0.25 µM IN/IBD for 2 h on ice, in the same binding reaction buffers as described above. IN/IBD or NC was then added to NC+RNA complex or IN/IBD + RNA complex, respectively, and incubated for another 2 h on ice in the same binding reaction buffers. The following protein concentrations were used: 0.2, 0.15, 0.1 or 0 µM for IN/IBD; 4 (to mimic near-physiological stoichiometry), 1, 0.7, 0.3 or 0 µM for NC. The addition of the second protein also led to dilution of RNA to 0.1 µM, preincubated NC to 1 µM, and preincubated IN/IBD to 0.2 µM.

After binding, half of the samples were used for SHAPE probing and half were used for UV crosslinking. For SHAPE probing, 10 µL NMIA (8 mM in DMSO) was added into 90 µL of each of the binding reactions. Control reactions without proteins contained either neat DMSO or NMIA (in DMSO). The SHAPE reactions were incubated at 37 °C for 45 min. For XL probing, each of the binding reactions (90 µL) was exposed to UV light (254 nm, total energy of 400 mJ/cm^2^) on ice in a UVP UV crosslinker CL-1000 model (Analytik Jena, Germany). Two control XL experiments were also performed without proteins: an RNA-only reaction was exposed to UV light to assess UV damage and a second RNA-only reaction was incubated on ice in the absence of UV irradiation. After SHAPE reaction or UV crosslinking radiation, all the samples were treated with 10 µL of 5% SDS and 1 µL of Proteinase K (New England Biolabs, Ipswich, USA), and incubated at 55 °C for 60 min. The RNAs were recovered by phenol–chloroform extraction and ethanol precipitation.

The primer extension and capillary electrophoresis (CE) experiments were performed as described previously [70, 78]. The sequence of the 5′-NED-labeled primer used in the primer extension reactions was: 5′-TAC CGA CGC TCT CGC ACC-3′ (from Applied Biosystems, Foster City). The CE experiments were performed in the Genomics Shared Resources facility (The Ohio State University). The raw data obtained from the CE analysis were analyzed using RiboCAT software [46]. The SHAPE data for RNA alone were used as pseudo-energy constraints for structure modeling using RNAstructure [45]. A helix file was generated in RNAstructure and loaded into XRNA (http://rna.ucsc.edu/rnacenter/xrna/xrna.html, UCSC), which was used to generate the secondary structure representation. The differences between the XL and SHAPE reactivity of the control and protein-bound samples were compared using an unpaired, two-tail student’s t-test. Absolute reactivity differences of ≥ 0.3 with a p value < 0.05 were considered to be statistically significant. Dose-dependent reactivity changes in the case of protein binding were an additional prerequisite for establishing significance. At least 3 independent trials were performed for all in vitro XL-SHAPE experiments. The raw data of all XL-SHAPE experiments can be found in Additional file 10.

### In virio SHAPE probing

HEK293T cells were propagated in humidified incubators at 37 °C in Dulbecco’s modified Eagles medium supplemented to contain 10% fetal bovine serum (FBS), 100 IU/mL penicillin, and 100 µg/mL streptomycin in the presence of 5% CO_2_. Cell-free HIV-1_NL4−3_ viral particles were produced by transfecting HEK293T cells in 15-cm cell culture plates with 30 µg pNL4-3 plasmid DNA [79] per plate using PolyJet DNA transfection reagent (SignaGen Laboratories). Where indicated, 10 µM BI-D was added to the cell media. Two days after transfection, 160 mL cell supernatants were filtered through 0.22 μm filters and pelleted by ultracentrifugation using a Beckman SW32-Ti rotor at 26,000 rpm for 2 h at 4 °C. Pellets were suspended in 160 µL 2× NMIA reaction buffer (50 mM HEPES (pH 8.0), 200 mM NaCl, 0.1 mM EDTA, 10% FBS). The resuspended virions were treated with NMIA (16 µL, 100 mM in DMSO) or vehicle control DMSO (16 µL) for 50 min at 37 °C. Viral gRNAs from both (+) and (−) NMIA treatments were gently extracted using Qiagen viral RNA miniprep Kit. The extracted viral RNA was treated with DNase (Life technologies, cat # AM1907) at 37 °C for 1 h. Viral RNA genomes were quantified by real-time reverse-transcription PCR (RT-PCR) as previously described [24].

Primer extension and CE analyses were performed with ~ 1.2 pmol of NMIA-modified or control RNA, as described above. The CE data were also analyzed as described above. Absolute SHAPE reactivity differences of ≥ 0.3 between BI-D-treated and DMSO-treated control samples were considered significant. Two independent trials were performed for in virio SHAPE experiments.

## Supplementary Information

**Additional file 1: Figure S1.** SHAPE reactivity-constrained lowest energy secondary structure of the 352-nt HIV-1 5′-UTR-ΔDIS RNA. The secondary structure model was generated by applying averaged normalized SHAPE reactivity from three independent trials as pseudo free-energy constraints. Nucleotides are colored in accordance to SHAPE reactivity as indicated in the key. Nucleotides that could not be analyzed are shown in grey. In this construct, the Psi DIS sequence (AAGCGCGCA) was replaced by a GAGA tetraloop (boxed). Nucleotide numbering is according to the WT HIV-1 5′-UTR sequence.

**Additional file 2: Figure S2.** Results of in vitro XL-SHAPE analysis of 352-nt HIV-1 5′-UTR-ΔDIS RNA in TAR/polyA region, upon binding to (A) NC; (B) IN/IBD; (C) NC + IN/IBD (NC incubated first) and (D) IN/IBD + NC (IN/IBD incubated first). Sites with decreased and increased SHAPE reactivity upon protein binding are indicated by green and red arrowheads, respectively. Identified crosslinking sites are labeled with stars. All identified sites had reactivity changes of ≥ 0.3 and p < 0.05 based on unpaired, two-tailed Student’s t-tests, compared with RNA alone control (A and B), RNA + NC (C), or RNA + IN/IBD (D). Results are based on the average of at least 3 independent experiments. Nucleotides that could not be analyzed are shown in grey.

**Additional file 3: Figure S3.** Results of in vitro XL-SHAPE analysis of 352-nt HIV-1 5′-UTR-ΔDIS RNA near the U5:AUG region, upon binding to (A) NC; (B) IN/IBD; (C) NC + IN/IBD (NC incubated first) and (D) IN/IBD + NC (IN/IBD incubated first). Sites with decreased and increased SHAPE reactivity upon protein binding are indicated by green and red arrowheads, respectively. Identified crosslinking sites are labeled with stars. All identified sites had reactivity changes of ≥ 0.3 and p < 0.05 based on unpaired, two-tailed Student’s t-tests, compared with RNA alone control (A and B), RNA + NC (C), or RNA + IN/IBD (D). Results are based on the average of at least 3 independent experiments. Nucleotides that could not be analyzed are shown in grey. Nucleotide numbering is according to the WT HIV-1 5′-UTR sequence.

**Additional file 4: Figure S4.** Results of in vitro XL-SHAPE analysis of 352-nt HIV-1 5′-UTR-ΔDIS RNA in the PBS/TLE region, upon binding to (A) NC; (B) IN/IBD; (C) NC + IN/IBD (NC incubated first) and (D) IN/IBD + NC (IN/IBD incubated first). Sites with decreased and increased SHAPE reactivity upon protein binding are indicated by green and red arrowheads, respectively. Identified crosslinking sites are labeled with stars. All identified sites had reactivity changes of ≥ 0.3 and p < 0.05 based on unpaired, two-tailed Student’s t-tests, compared with RNA alone control (A and B), RNA + NC (C), or RNA + IN/IBD (D). Results are based on the average of at least 3 independent experiments.

**Additional file 5: Figure S5.** Results of in vitro XL-SHAPE analysis of 352-nt HIV-1 5′-UTR-ΔDIS RNA in the Psi region, upon binding to (A) NC; (B) IN/IBD; (C) NC + IN/IBD (NC incubated first) and (D) IN/IBD + NC (IN/IBD incubated first). Sites with decreased and increased SHAPE reactivity upon protein binding are indicated by green and red arrowheads, respectively. Identified crosslinking sites are labeled with stars. All identified sites had reactivity changes of ≥ 0.3 and p < 0.05 based on unpaired, two-tailed Student’s t-tests, compared with RNA alone control (A and B), RNA + NC (C), or RNA + IN/IBD (D). Results are based on the average of at least 3 independent experiments. Nucleotides that could not be analyzed are shown in grey. The Psi DIS sequence (AAGCGCGCA) was replaced by a GAGA tetraloop (boxed). Nucleotide numbering is according to the WT HIV-1 5′-UTR sequence.

**Additional file 6: Figure S6.** XL-SHAPE analysis of IBD binding to the 352-nt HIV-1 5′-UTR-ΔDIS RNA. Sites with decreased and increased SHAPE reactivity upon protein binding are indicated by green and red arrowheads, respectively. All identified sites have reactivity changes of ≥ 0.3 and p < 0.05 based on unpaired, two-tailed Student’s t-tests, compared with the no protein control. Results are based on the average of at least 3 independent experiments. Other information is as noted in the legend to Fig. S1.

**Additional file 7: Figure S7.** XL-SHAPE analysis of IN (R269A, K273A) binding to the 352-nt HIV-1 5′-UTR-ΔDIS RNA. Sites with decreased and increased SHAPE reactivity upon protein binding are indicated by green and red arrowheads, respectively. All identified sites have reactivity changes of ≥ 0.3 and p < 0.05 based on unpaired, two-tailed Student’s t-tests, compared with the no protein control. Results are based on the average of at least 3 independent experiments. Other information is as noted in the legend to Fig. S1.

**Additional file 8: Figure S8.** XL-SHAPE analysis of near-physiological stoichiometry of NC binding to the HIV-1 5′UTR-ΔDIS/IN/IBD complex. Sites with decreased and increased SHAPE reactivity upon protein binding are indicated by green and red arrowheads, respectively. Identified crosslinking sites are labeled with stars. All identified sites have reactivity changes of ≥ 0.3 and p < 0.05 based on unpaired, two-tailed Student’s t-tests, compared with the RNA + IN/IBD control. Results are based on the average of at least 2 independent experiments. Other information is as noted in the legend to Fig. S1.

**Additional file 9: Figure S9.** In virio SHAPE reactivity-constrained lowest energy secondary structure of the HIV-1 5′-UTR after treatment of cells with DMSO. The secondary structure model was generated by applying averaged normalized SHAPE reactivity from two independent trials as pseudo free-energy constraints. Nucleotides are colored according to SHAPE reactivity as indicated in the key. Nucleotides that could not be analyzed are shown in grey. The tRNA^Lys,3^ annealing site is indicated by a black line.

**Additional file 10.** Raw data files for all of the XL-SHAPE studies reported here.

## Data Availability

The datasets used and/or analyzed during the current study are available in Additional file 10.

## Abbreviations

IN: Integrase
gRNA: Genomic RNA
NC: Nucleocapsid
XL-SHAPE: Crosslinking coupled selective 2′-hydroxyl acylation analyzed by primer extension
5′-UTR: 5′-Untranslated region
Psi: Packaging signal
TAR: Transactivation response element
ALLINI: Allosteric IN inhibitor
CA: Capsid
vRNP: Viral ribonucleoprotein complex
RT: Reverse transcriptase
PBS: Primer binding site
DIS: D imerization initiation signal
IBD: IN-binding domain
LEDGF: Lens-epithelium-derived-growth-factor
TLE: tRNA-like element
DMSO: Dimethyl sulfoxide
HEPES: 4-(2-Hydroxyethyl)-1-piperazineethanesulfonic acid
BME: 2-Mercaptoethanol
NMIA: *N*-Methylisatoic anhydride
CE: Capillary electrophoresis
FBS: Fetal bovine serum
RT-PCR: Real-time reverse-transcription PCR

## References

[1] Freed EO (2015). HIV-1 assembly, release and maturation. Nat Rev Microbiol.

[2] Sundquist WI, Krausslich HG (2012). HIV-1 assembly, budding, and maturation. Cold Spring Harbor Perspect Med.

[3] Pornillos O, Ganser-Pornillos BK (2019). Maturation of retroviruses. Curr Opin Virol.

[4] Lesbats P, Engelman AN, Cherepanov P, Retroviral (2016). DNA integration. Chem Rev.

[5] Engelman A (1999). In vivo analysis of retroviral integrase structure and function. Adv Virus Res.

[6] Elliott JL, Kutluay SB (2020). Going beyond Integration: the emerging role of HIV-1 integrase in virion morphogenesis. Viruses.

[7] Engelman A, Englund G, Orenstein JM, Martin MA, Craigie R (1995). Multiple effects of mutations in human immunodeficiency virus type 1 integrase on viral replication. J Virol.

[8] Jenkins TM, Engelman A, Ghirlando R, Craigie R (1996). A soluble active mutant of HIV-1 integrase: involvement of both the core and carboxyl-terminal domains in multimerization. J Biol Chem.

[9] Nakamura T, Masuda T, Goto T, Sano K, Nakai M, Harada S (1997). Lack of infectivity of HIV-1 integrase zinc finger-like domain mutant with morphologically normal maturation. Biochem Biophys Res Commun.

[10] Shin CG, Taddeo B, Haseltine WA, Farnet CM (1994). Genetic analysis of the human immunodeficiency virus type 1 integrase protein. J Virol.

[11] Fontana J, Jurado KA, Cheng N, Ly NL, Fuchs JR, Gorelick RJ (2015). Distribution and redistribution of HIV-1 nucleocapsid protein in immature, mature, and integrase-inhibited virions: a role for integrase in maturation. J Virol.

[12] Kessl JJ, Kutluay SB, Townsend D, Rebensburg S, Slaughter A, Larue RC (2016). HIV-1 integrase binds the viral RNA genome and is essential during virion morphogenesis. Cell.

[13] Elliott JL, Eschbach JE, Koneru PC, Li W, Puray-Chavez M, Townsend D (2020). Integrase–RNA interactions underscore the critical role of integrase in HIV-1 virion morphogenesis. eLife.

[14] Quillent C, Borman AM, Paulous S, Dauguet C, Clavel F (1996). Extensive regions of pol are required for efficient human immunodeficiency virus polyprotein processing and particle maturation. Virology.

[15] Le Rouzic E, Bonnard D, Chasset S, Bruneau JM, Chevreuil F, Le Strat F (2013). Dual inhibition of HIV-1 replication by integrase-LEDGF allosteric inhibitors is predominant at the post-integration stage. Retrovirology.

[16] Kessl JJ, Jena N, Koh Y, Taskent-Sezgin H, Slaughter A, Feng L (2012). Multimode, cooperative mechanism of action of allosteric HIV-1 integrase inhibitors. J Biol Chem.

[17] Gupta K, Brady T, Dyer BM, Malani N, Hwang Y, Male F (2014). Allosteric inhibition of human immunodeficiency virus integrase: late block during viral replication and abnormal multimerization involving specific protein domains. J Biol Chem.

[18] Fader LD, Malenfant E, Parisien M, Carson R, Bilodeau F, Landry S (2014). Discovery of BI 224436, a noncatalytic site integrase inhibitor (NCINI) of HIV-1. ACS Med Chem Lett.

[19] Christ F, Voet A, Marchand A, Nicolet S, Desimmie BA, Marchand D (2010). Rational design of small-molecule inhibitors of the LEDGF/p75-integrase interaction and HIV replication. Nat Chem Biol.

[20] Wilson TA, Koneru PC, Rebensburg SV, Lindenberger JJ, Kobe MJ, Cockroft NT (2019). An isoquinoline scaffold as a novel class of allosteric HIV-1 integrase inhibitors. ACS Med Chem Lett.

[21] Deng N, Hoyte A, Mansour YE, Mohamed MS, Fuchs JR, Engelman AN (2016). Allosteric HIV-1 integrase inhibitors promote aberrant protein multimerization by directly mediating inter-subunit interactions: structural and thermodynamic modeling studies. Protein Sci.

[22] Feng L, Sharma A, Slaughter A, Jena N, Koh Y, Shkriabai N (2013). The A128T resistance mutation reveals aberrant protein multimerization as the primary mechanism of action of allosteric HIV-1 integrase inhibitors. J Biol Chem.

[23] Desimmie BA, Schrijvers R, Demeulemeester J, Borrenberghs D, Weydert C, Thys W (2013). LEDGINs inhibit late stage HIV-1 replication by modulating integrase multimerization in the virions. Retrovirology.

[24] Jurado KA, Wang H, Slaughter A, Feng L, Kessl JJ, Koh Y (2013). Allosteric integrase inhibitor potency is determined through the inhibition of HIV-1 particle maturation. Proc Natl Acad Sci USA.

[25] Tsiang M, Jones GS, Niedziela-Majka A, Kan E, Lansdon EB, Huang W (2012). New class of HIV-1 integrase (IN) inhibitors with a dual mode of action. J Biol Chem.

[26] Sharma A, Slaughter A, Jena N, Feng L, Kessl JJ, Fadel HJ (2014). A new class of multimerization selective inhibitors of HIV-1 integrase. PLoS Pathog.

[27] Balakrishnan M, Yant SR, Tsai L, O’Sullivan C, Bam RA, Tsai A (2013). Non-catalytic site HIV-1 integrase inhibitors disrupt core maturation and induce a reverse transcription block in target cells. PLoS ONE.

[28] Bonnard D, Le Rouzic E, Eiler S, Amadori C, Orlov I, Bruneau JM (2018). Structure-function analyses unravel distinct effects of allosteric inhibitors of HIV-1 integrase on viral maturation and integration. J Biol Chem.

[29] Madison MK, Lawson DQ, Elliott J, Ozanturk AN, Koneru PC, Townsend D (2017). Allosteric HIV-1 integrase inhibitors lead to premature degradation of the viral RNA genome and integrase in target cells. J Virol.

[30] Koneru PC, Francis AC, Deng N, Rebensburg SV, Hoyte AC, Lindenberger J (2019). HIV-1 integrase tetramers are the antiviral target of pyridine-based allosteric integrase inhibitors. eLife.

[31] Lu K, Heng X, Summers MF (2011). Structural determinants and mechanism of HIV-1 genome packaging. J Mol Biol.

[32] Kuzembayeva M, Dilley K, Sardo L, Hu WS (2014). Life of psi: how full-length HIV-1 RNAs become packaged genomes in the viral particles. Virology.

[33] Abd El-Wahab EW, Smyth RP, Mailler E, Bernacchi S, Vivet-Boudou V, Hijnen M (2014). Specific recognition of the HIV-1 genomic RNA by the Gag precursor. Nat Commun.

[34] Webb JA, Jones CP, Parent LJ, Rouzina I, Musier-Forsyth K (2013). Distinct binding interactions of HIV-1 Gag to Psi and non-Psi RNAs: implications for viral genomic RNA packaging. RNA.

[35] Sarni S, Biswas B, Liu S, Olson ED, Kitzrow JP, Rein A (2020). HIV-1 Gag protein with or without p6 specifically dimerizes on the viral RNA packaging signal. J Biol Chem.

[36] Smyth RP, Smith MR, Jousset AC, Despons L, Laumond G, Decoville T (2018). In cell mutational interference mapping experiment (in cell MIME) identifies the 5′ polyadenylation signal as a dual regulator of HIV-1 genomic RNA production and packaging. Nucleic Acids Res.

[37] Nikolaitchik OA, Somoulay X, Rawson JMO, Yoo JA, Pathak VK, Hu WS (2020). Unpaired guanosines in the 5′ untranslated region of HIV-1 RNA Act synergistically to mediate genome packaging. J Virol.

[38] Wilkinson KA, Gorelick RJ, Vasa SM, Guex N, Rein A, Mathews DH (2008). High-throughput SHAPE analysis reveals structures in HIV-1 genomic RNA strongly conserved across distinct biological states. PLoS Biol.

[39] Kutluay SB, Zang T, Blanco-Melo D, Powell C, Jannain D, Errando M (2014). Global changes in the RNA binding specificity of HIV-1 gag regulate virion genesis. Cell.

[40] Kenyon JC, Prestwood LJ, Lever AM (2015). A novel combined RNA-protein interaction analysis distinguishes HIV-1 Gag protein binding sites from structural change in the viral RNA leader. Sci Rep.

[41] Jones CP, Cantara WA, Olson ED, Musier-Forsyth K (2014). Small-angle X-ray scattering-derived structure of the HIV-1 5′ UTR reveals 3D tRNA mimicry. Proc Natl Acad Sci USA.

[42] Masuda T, Sato Y, Huang YL, Koi S, Takahata T, Hasegawa A (2015). Fate of HIV-1 cDNA intermediates during reverse transcription is dictated by transcription initiation site of virus genomic RNA. Sci Rep.

[43] Kharytonchyk S, Monti S, Smaldino PJ, Van V, Bolden NC, Brown JD (2016). Transcriptional start site heterogeneity modulates the structure and function of the HIV-1 genome. Proc Natl Acad Sci.

[44] Brown JD, Kharytonchyk S, Chaudry I, Iyer AS, Carter H, Becker G (2020). Structural basis for transcriptional start site control of HIV-1 RNA fate.

[45] Reuter JS, Mathews DH (2010). RNAstructure: software for RNA secondary structure prediction and analysis. BMC Bioinform.

[46] Cantara WA, Hatterschide J, Wu W, Musier-Forsyth K (2017). RiboCAT: a new capillary electrophoresis data analysis tool for nucleic acid probing. RNA.

[47] Kessl JJ, Li M, Ignatov M, Shkriabai N, Eidahl JO, Feng L (2011). FRET analysis reveals distinct conformations of IN tetramers in the presence of viral DNA or LEDGF/p75. Nucleic Acids Res.

[48] McKee CJ, Kessl JJ, Shkriabai N, Dar MJ, Engelman A, Kvaratskhelia M (2008). Dynamic modulation of HIV-1 integrase structure and function by cellular lens epithelium-derived growth factor (LEDGF) protein. J Biol Chem.

[49] Llano M, Saenz DT, Meehan A, Wongthida P, Peretz M, Walker WH (2006). An essential role for LEDGF/p75 in HIV integration. Science.

[50] Engelman A, Liu Y, Chen H, Farzan M, Dyda F (1997). Structure-based mutagenesis of the catalytic domain of human immunodeficiency virus type 1 integrase. J Virol.

[51] Bannwarth S, Gatignol A (2005). HIV-1 TAR RNA: the target of molecular interactions between the virus and its host. Curr HIV Res.

[52] Fisher RJ, Rein A, Fivash M, Urbaneja MA, Casas-Finet JR, Medaglia M (1998). Sequence-specific binding of human immunodeficiency virus type 1 nucleocapsid protein to short oligonucleotides. J Virol.

[53] Vuilleumier C, Bombarda E, Morellet N, Gerard D, Roques BP, Mely Y (1999). Nucleic acid sequence discrimination by the HIV-1 nucleocapsid protein NCp7: a fluorescence study. Biochemistry.

[54] Keane SC, Heng X, Lu K, Kharytonchyk S, Ramakrishnan V, Carter G (2015). RNA structure. Structure of the HIV-1 RNA packaging signal.

[55] Mak J, Kleiman L (1997). Primer tRNAs for reverse transcription. J Virol.

[56] Jones CP, Saadatmand J, Kleiman L, Musier-Forsyth K (2013). Molecular mimicry of human tRNALys anti-codon domain by HIV-1 RNA genome facilitates tRNA primer annealing. RNA.

[57] Liu S, Comandur R, Jones CP, Tsang P, Musier-Forsyth K (2016). Anticodon-like binding of the HIV-1 tRNA-like element to human lysyl-tRNA synthetase. RNA.

[58] Lever A, Gottlinger H, Haseltine W, Sodroski J (1989). Identification of a sequence required for efficient packaging of human immunodeficiency virus type 1 RNA into virions. J Virol.

[59] Gorelick RJ, Nigida SM, Bess JW, Arthur LO, Henderson LE, Rein A (1990). Noninfectious human immunodeficiency virus type 1 mutants deficient in genomic RNA. J Virol.

[60] Aldovini A, Young RA (1990). Mutations of RNA and protein sequences involved in human immunodeficiency virus type 1 packaging result in production of noninfectious virus. J Virol.

[61] Bieniasz P, Telesnitsky A (2018). Multiple, switchable protein: RNA interactions regulate human immunodeficiency virus type 1 assembly. Annu Rev Virol.

[62] Olson ED, Musier-Forsyth K (2018). Retroviral Gag protein–RNA interactions implications for specific genomic RNA packaging and virion assembly. Seminars in cell & developmental biology.

[63] Rein A (2019). RNA packaging in HIV. Trends Microbiol.

[64] Briggs JA, Simon MN, Gross I, Krausslich HG, Fuller SD, Vogt VM (2004). The stoichiometry of Gag protein in HIV-1. Nat Struct Mol Biol.

[65] Christensen DE, Ganser-Pornillos BK, Johnson JS, Pornillos O, Sundquist WI (2020). Reconstitution and visualization of HIV-1 capsid-dependent replication and integration in vitro.

[66] De Guzman RN, Wu ZR, Stalling CC, Pappalardo L, Borer PN, Summers MF (1998). Structure of the HIV-1 nucleocapsid protein bound to the SL3 psi-RNA recognition element.

[67] Ding P, Kharytonchyk S, Waller A, Mbaekwe U, Basappa S, Kuo N (2020). Identification of the initial nucleocapsid recognition element in the HIV-1 RNA packaging signal. Proc Natl Acad Sci USA.

[68] Rye-McCurdy T, Olson ED, Liu S, Binkley C, Reyes JP, Thompson BR (2016). Functional equivalence of retroviral ma domains in facilitating Psi RNA binding specificity by Gag. Viruses.

[69] Smyth RP, Despons L, Huili G, Bernacchi S, Hijnen M, Mak J (2015). Mutational interference mapping experiment (MIME) for studying RNA structure and function. Nat Methods.

[70] Liu S, Kaddis Maldonado R, Rye-McCurdy T, Binkley C, Bah A, Chen EC (2020). Rous sarcoma virus genomic RNA dimerization capability in vitro is not a prerequisite for viral infectivity. Viruses.

[71] Seif E, Niu M, Kleiman L (2015). In virio SHAPE analysis of tRNA(Lys3) annealing to HIV-1 genomic RNA in wild type and protease-deficient virus. Retrovirology.

[72] Jalalirad M, Laughrea M (2010). Formation of immature and mature genomic RNA dimers in wild-type and protease-inactive HIV-1: differential roles of the Gag polyprotein, nucleocapsid proteins NCp15, NCp9, NCp7, and the dimerization initiation site. Virology.

[73] Song R, Kafaie J, Yang L, Laughrea M (2007). HIV-1 viral RNA is selected in the form of monomers that dimerize in a three-step protease-dependent process; the DIS of stem-loop 1 initiates viral RNA dimerization. J Mol Biol.

[74] Cherepanov P, Sun ZY, Rahman S, Maertens G, Wagner G, Engelman A (2005). Solution structure of the HIV-1 integrase-binding domain in LEDGF/p75. Nat Struct Mol Biol.

[75] Passos DO, Li M, Yang R, Rebensburg SV, Ghirlando R, Jeon Y (2017). Cryo-EM structures and atomic model of the HIV-1 strand transfer complex intasome.

[76] Cherepanov P (2007). LEDGF/p75 interacts with divergent lentiviral integrases and modulates their enzymatic activity in vitro. Nucleic Acids Res.

[77] Milligan JF, Groebe DR, Witherell GW, Uhlenbeck OC (1987). Oligoribonucleotide synthesis using T7 RNA polymerase and synthetic DNA templates. Nucleic Acids Res.

[78] Wu W, Hatterschide J, Syu YC, Cantara WA, Blower RJ, Hanson HM (2018). Human T-cell leukemia virus type 1 Gag domains have distinct RNA-binding specificities with implications for RNA packaging and dimerization. J Biol Chem.

[79] Adachi A, Gendelman HE, Koenig S, Folks T, Willey R, Rabson A (1986). Production of acquired immunodeficiency syndrome-associated retrovirus in human and nonhuman cells transfected with an infectious molecular clone. J Virol.

