# Supplementary figures and images for: HIV-1 integrase binding to genomic RNA 5′-UTR induces local structural changes in vitro and in virio
Source: Retrovirology. 2021 Nov 22;18:37. doi: 10.1186/s12977-021-00582-0 (PMC8609798; doi:10.1186/s12977-021-00582-0)

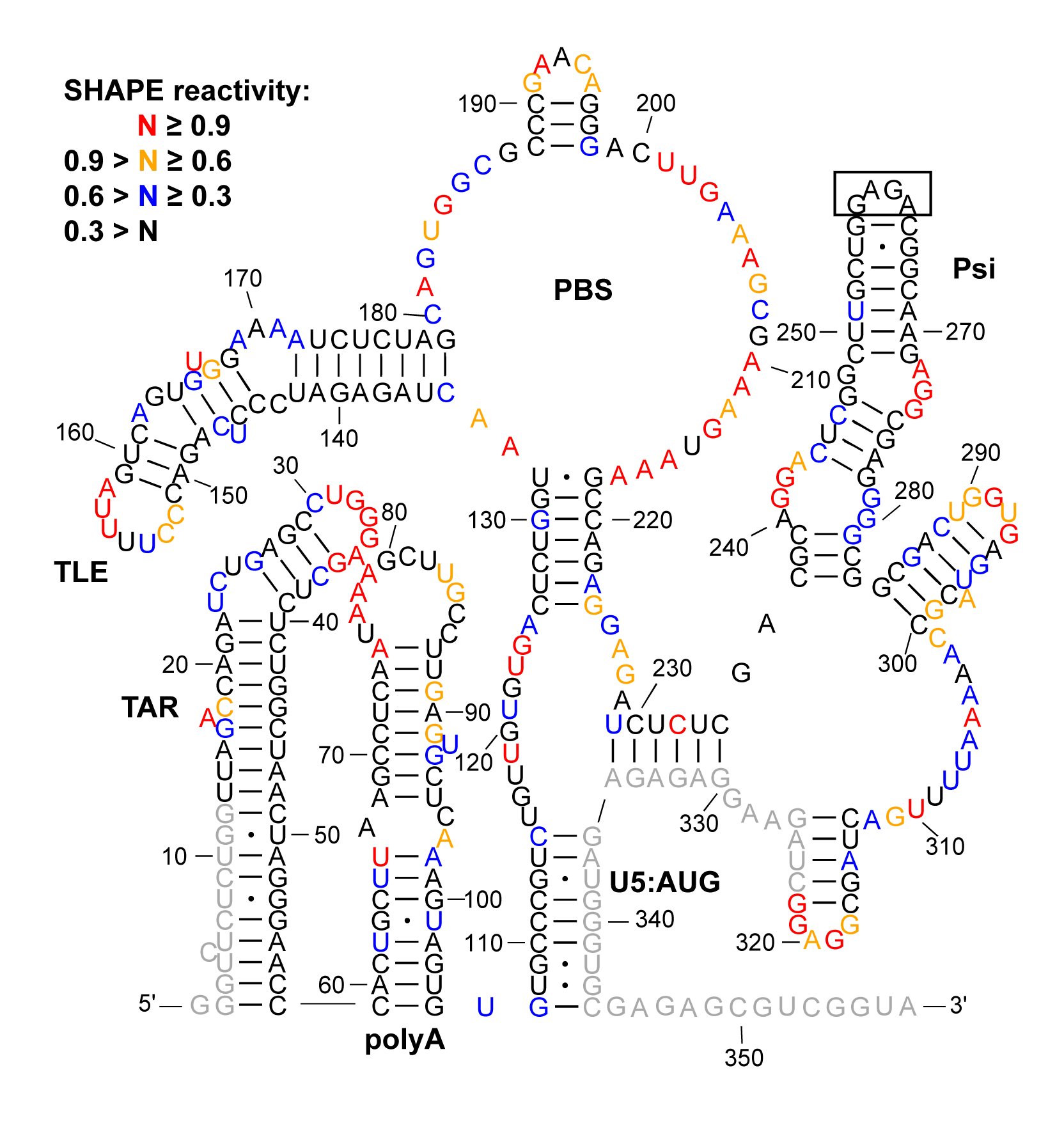

Supplement: Supplementary file 1 — Additional file 1: Figure S1. SHAPE reactivity-constrained lowest energy secondary structure of the 352-nt HIV-1 5′-UTR-ΔDIS RNA. The secondary structure model was generated by applying averaged normalized SHAPE reactivity from three independent trials as pseudo free-energy constraints. Nucleotides are colored in accordance to SHAPE reactivity as indicated in the key. Nucleotides that could not be analyzed are shown in grey. In this construct, the Psi DIS sequence (AAGCGCGCA) was replaced by a GAGA tetraloop (boxed). Nucleotide numbering is according to the WT HIV-1 5′-UTR sequence. [file 12977_2021_582_MOESM1_ESM.tif]

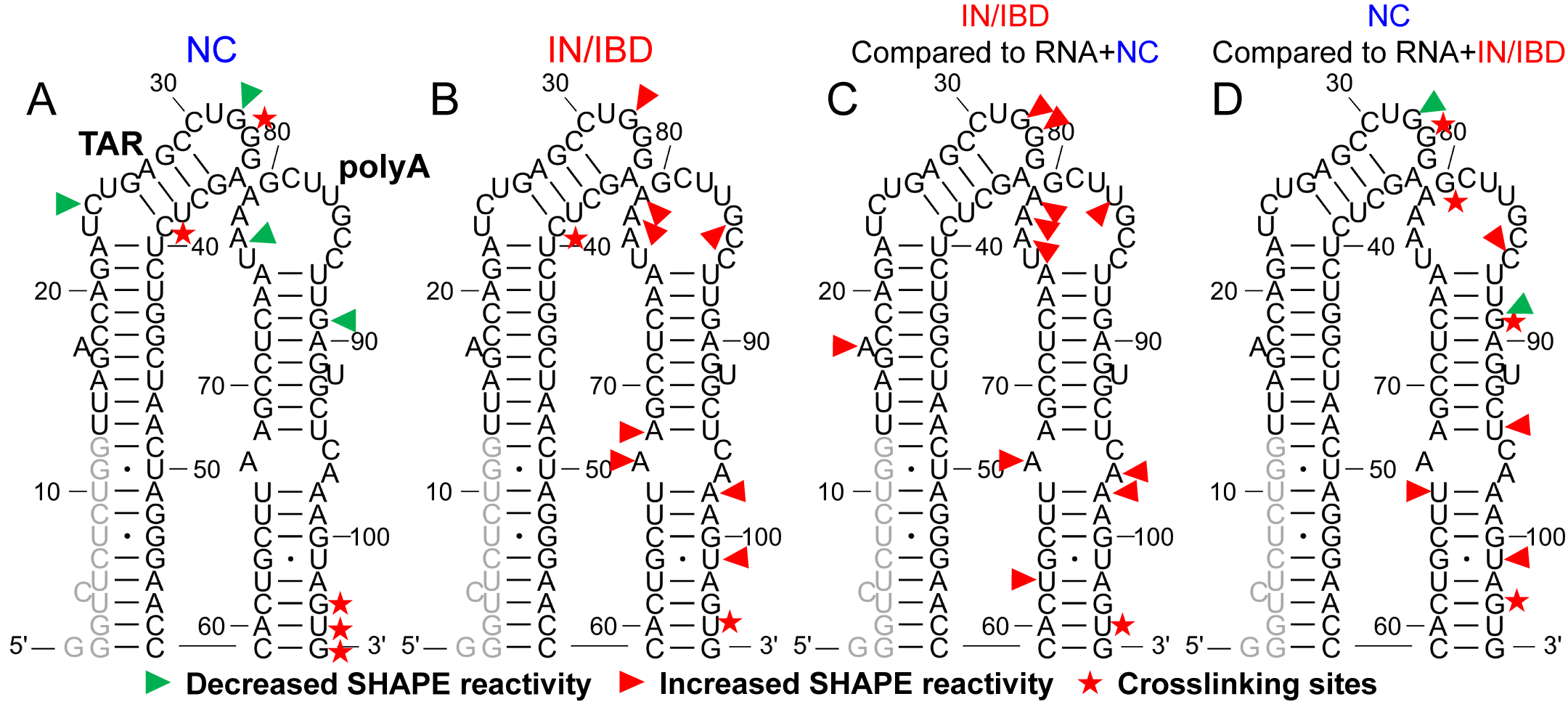

Supplement: Supplementary file 2 — Additional file 2: Figure S2. Results of in vitro XL-SHAPE analysis of 352-nt HIV-1 5′-UTR-ΔDIS RNA in TAR/polyA region, upon binding to (A) NC; (B) IN/IBD; (C) NC + IN/IBD (NC incubated first) and (D) IN/IBD + NC (IN/IBD incubated first). Sites with decreased and increased SHAPE reactivity upon protein binding are indicated by green and red arrowheads, respectively. Identified crosslinking sites are labeled with stars. All identified sites had reactivity changes of ≥ 0.3 and p < 0.05 based on unpaired, two-tailed Student’s t-tests, compared with RNA alone control (A and B), RNA + NC (C), or RNA + IN/IBD (D). Results are based on the average of at least 3 independent experiments. Nucleotides that could not be analyzed are shown in grey. [file 12977_2021_582_MOESM2_ESM.tif]

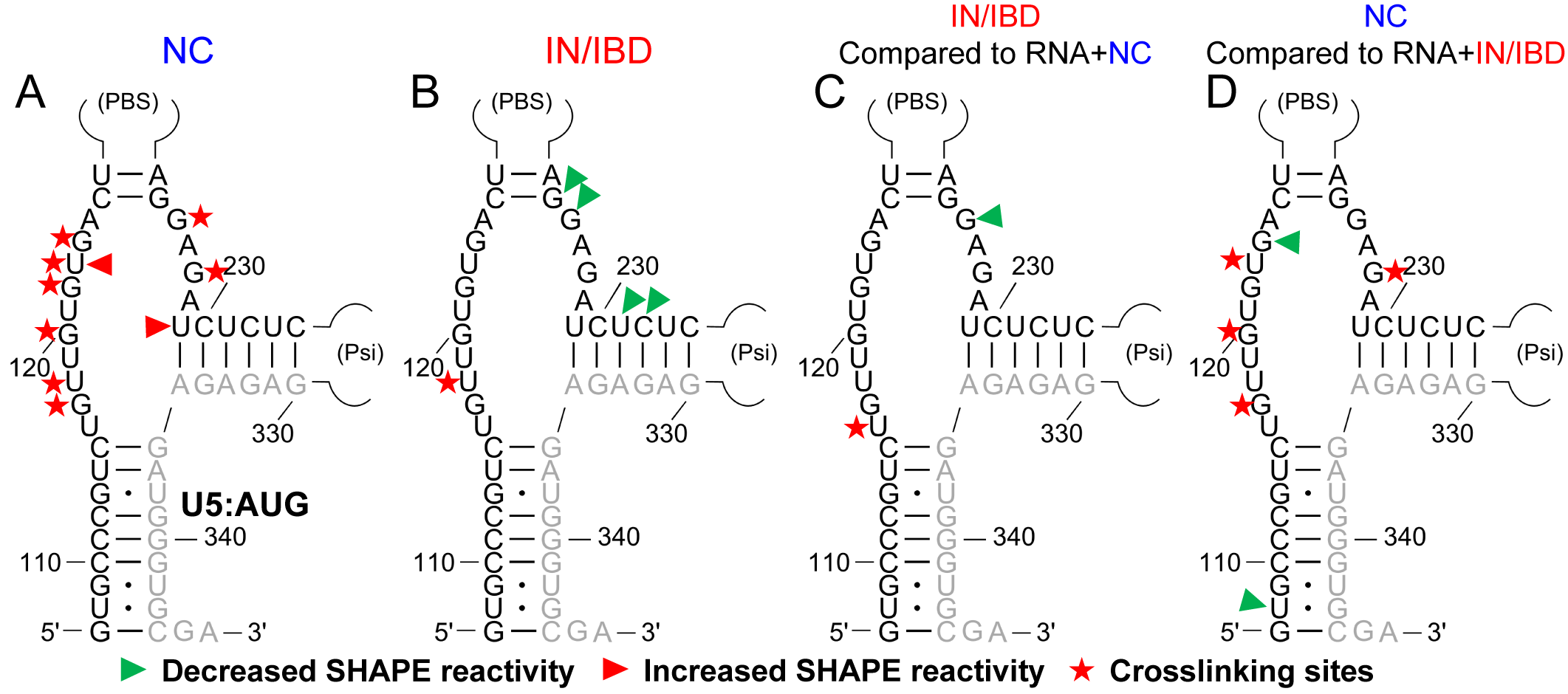

Supplement: Supplementary file 3 — Additional file 3: Figure S3. Results of in vitro XL-SHAPE analysis of 352-nt HIV-1 5′-UTR-ΔDIS RNA near the U5:AUG region, upon binding to (A) NC; (B) IN/IBD; (C) NC + IN/IBD (NC incubated first) and (D) IN/IBD + NC (IN/IBD incubated first). Sites with decreased and increased SHAPE reactivity upon protein binding are indicated by green and red arrowheads, respectively. Identified crosslinking sites are labeled with stars. All identified sites had reactivity changes of ≥ 0.3 and p < 0.05 based on unpaired, two-tailed Student’s t-tests, compared with RNA alone control (A and B), RNA + NC (C), or RNA + IN/IBD (D). Results are based on the average of at least 3 independent experiments. Nucleotides that could not be analyzed are shown in grey. Nucleotide numbering is according to the WT HIV-1 5′-UTR sequence. [file 12977_2021_582_MOESM3_ESM.tif]

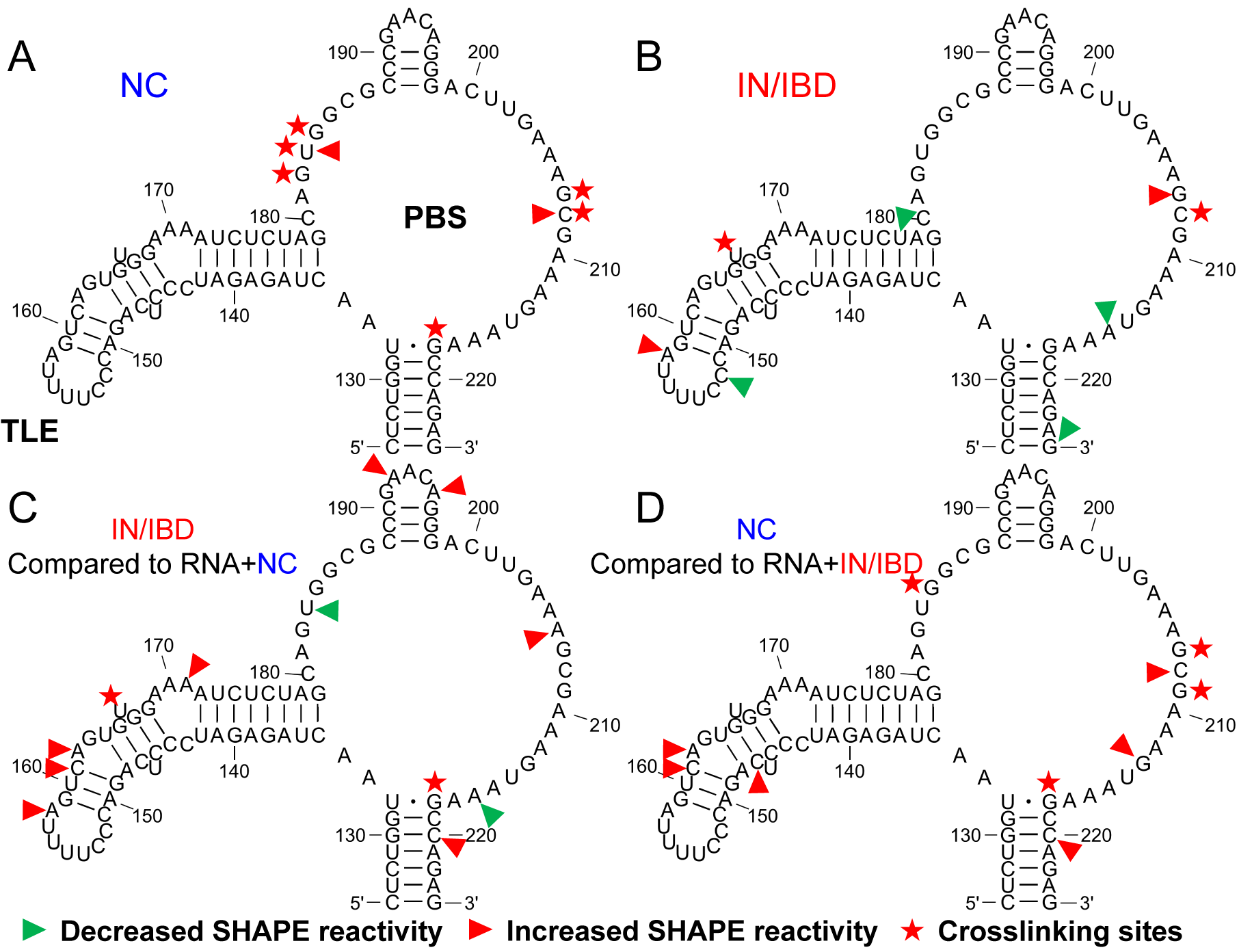

Supplement: Supplementary file 4 — Additional file 4: Figure S4. Results of in vitro XL-SHAPE analysis of 352-nt HIV-1 5′-UTR-ΔDIS RNA in the PBS/TLE region, upon binding to (A) NC; (B) IN/IBD; (C) NC + IN/IBD (NC incubated first) and (D) IN/IBD + NC (IN/IBD incubated first). Sites with decreased and increased SHAPE reactivity upon protein binding are indicated by green and red arrowheads, respectively. Identified crosslinking sites are labeled with stars. All identified sites had reactivity changes of ≥ 0.3 and p < 0.05 based on unpaired, two-tailed Student’s t-tests, compared with RNA alone control (A and B), RNA + NC (C), or RNA + IN/IBD (D). Results are based on the average of at least 3 independent experiments. [file 12977_2021_582_MOESM4_ESM.tif]

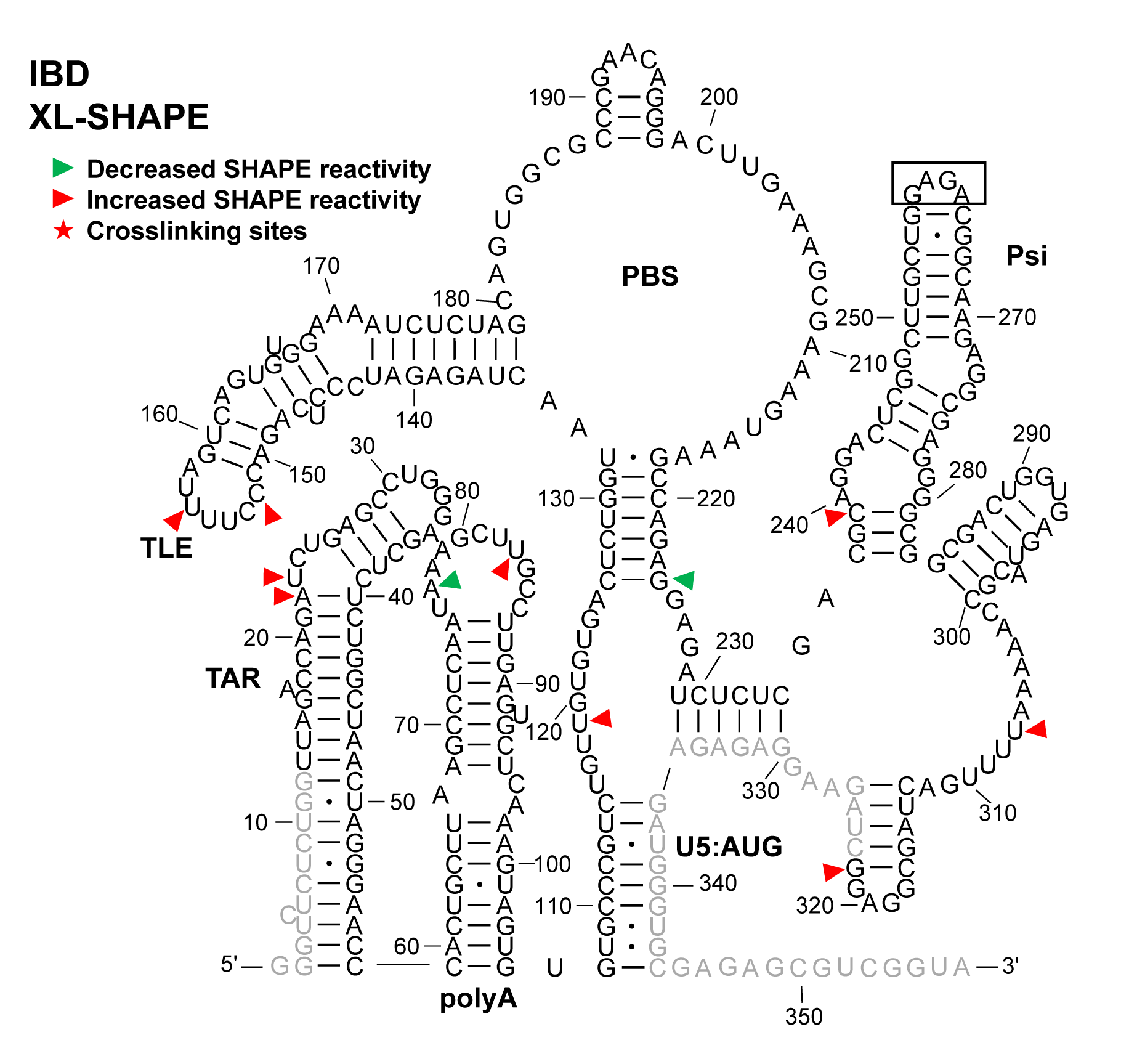

Supplement: Supplementary file 5 — Additional file 5: Figure S5. Results of in vitro XL-SHAPE analysis of 352-nt HIV-1 5′-UTR-ΔDIS RNA in the Psi region, upon binding to (A) NC; (B) IN/IBD; (C) NC + IN/IBD (NC incubated first) and (D) IN/IBD + NC (IN/IBD incubated first). Sites with decreased and increased SHAPE reactivity upon protein binding are indicated by green and red arrowheads, respectively. Identified crosslinking sites are labeled with stars. All identified sites had reactivity changes of ≥ 0.3 and p < 0.05 based on unpaired, two-tailed Student’s t-tests, compared with RNA alone control (A and B), RNA + NC (C), or RNA + IN/IBD (D). Results are based on the average of at least 3 independent experiments. Nucleotides that could not be analyzed are shown in grey. The Psi DIS sequence (AAGCGCGCA) was replaced by a GAGA tetraloop (boxed). Nucleotide numbering is according to the WT HIV-1 5′-UTR sequence. [file 12977_2021_582_MOESM5_ESM.tif]

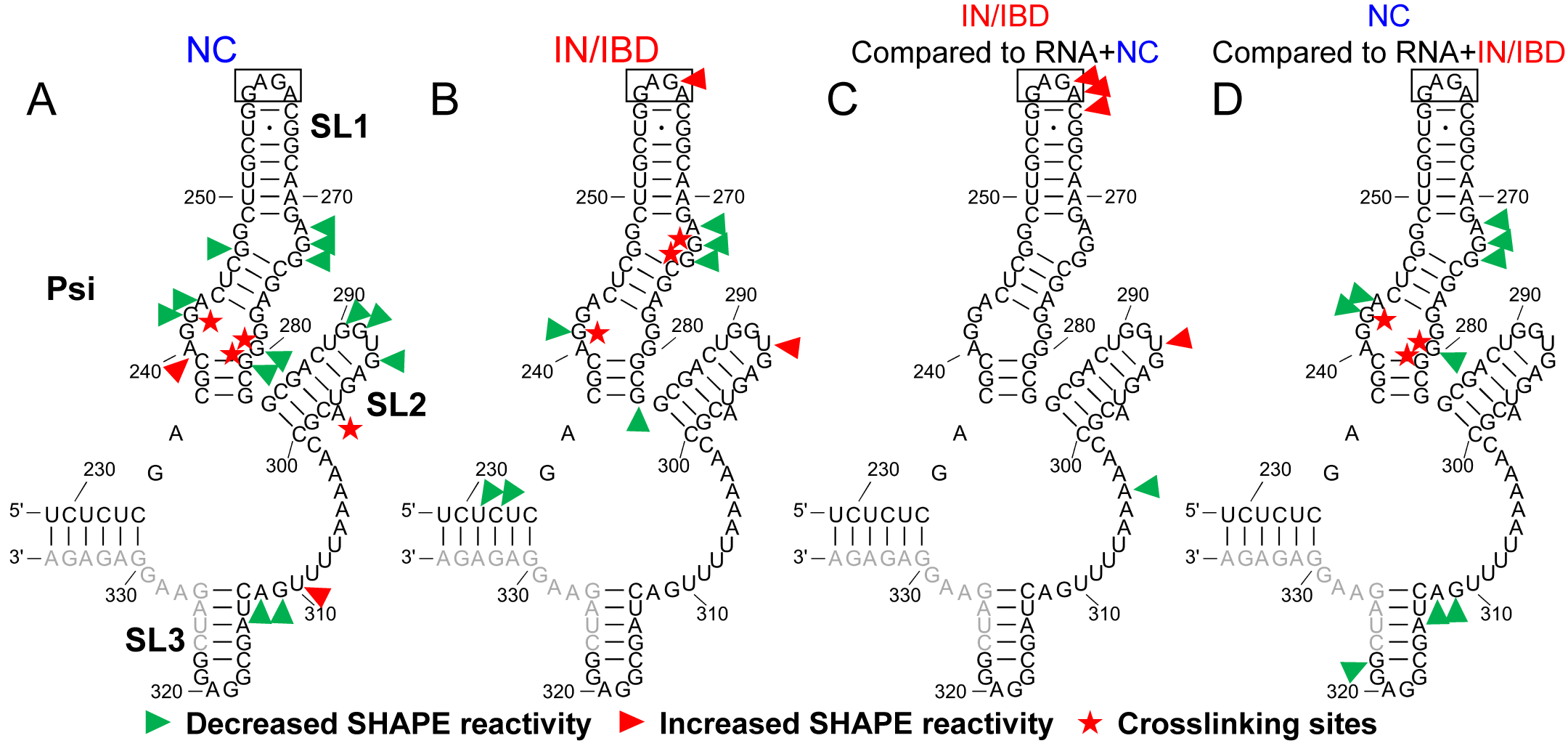

Supplement: Supplementary file 6 — Additional file 6: Figure S6. XL-SHAPE analysis of IBD binding to the 352-nt HIV-1 5′-UTR-ΔDIS RNA. Sites with decreased and increased SHAPE reactivity upon protein binding are indicated by green and red arrowheads, respectively. All identified sites have reactivity changes of ≥ 0.3 and p < 0.05 based on unpaired, two-tailed Student’s t-tests, compared with the no protein control. Results are based on the average of at least 3 independent experiments. Other information is as noted in the legend to Fig. S1. [file 12977_2021_582_MOESM6_ESM.tif]

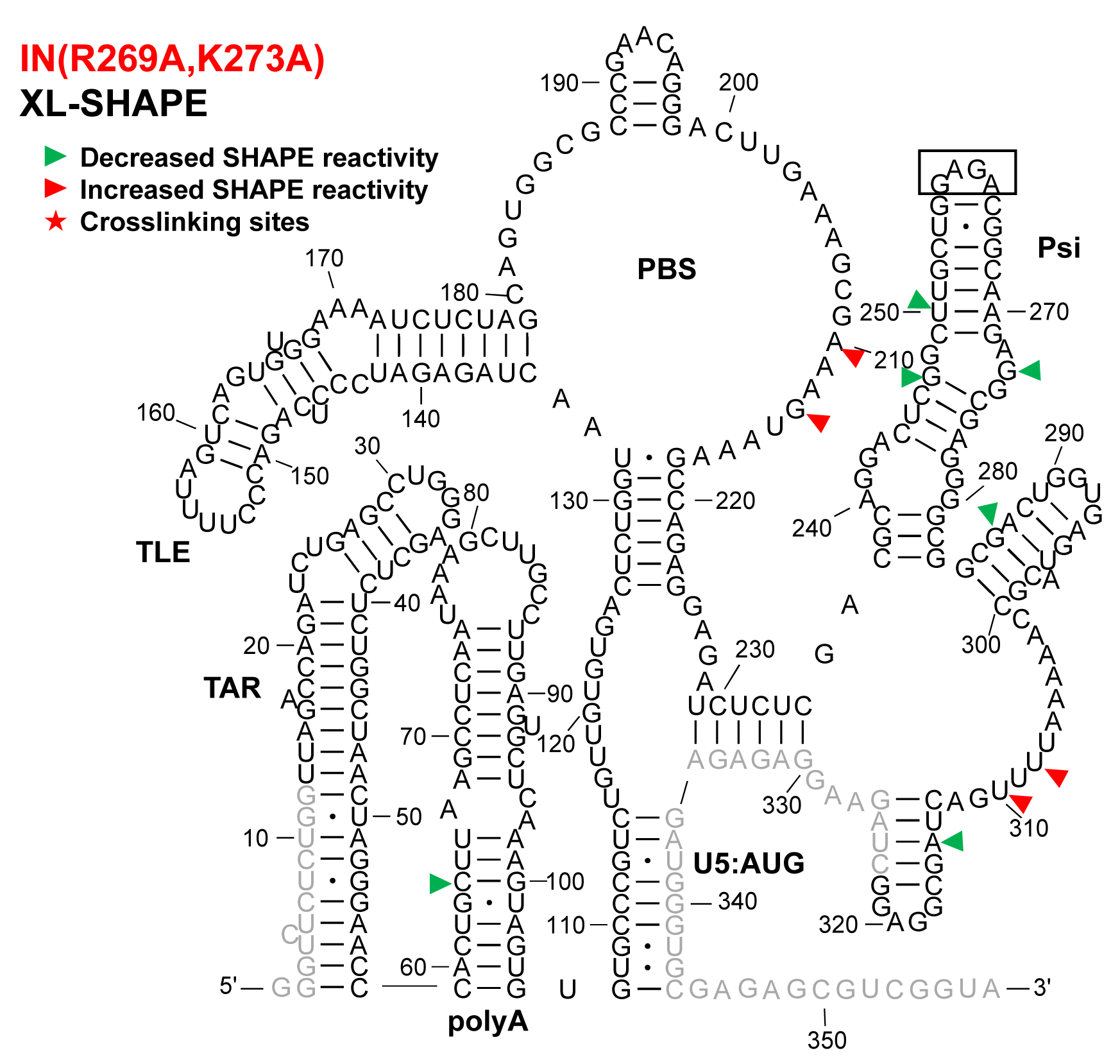

Supplement: Supplementary file 7 — Additional file 7: Figure S7. XL-SHAPE analysis of IN (R269A, K273A) binding to the 352-nt HIV-1 5′-UTR-ΔDIS RNA. Sites with decreased and increased SHAPE reactivity upon protein binding are indicated by green and red arrowheads, respectively. All identified sites have reactivity changes of ≥ 0.3 and p < 0.05 based on unpaired, two-tailed Student’s t-tests, compared with the no protein control. Results are based on the average of at least 3 independent experiments. Other information is as noted in the legend to Fig. S1. [file 12977_2021_582_MOESM7_ESM.tif]

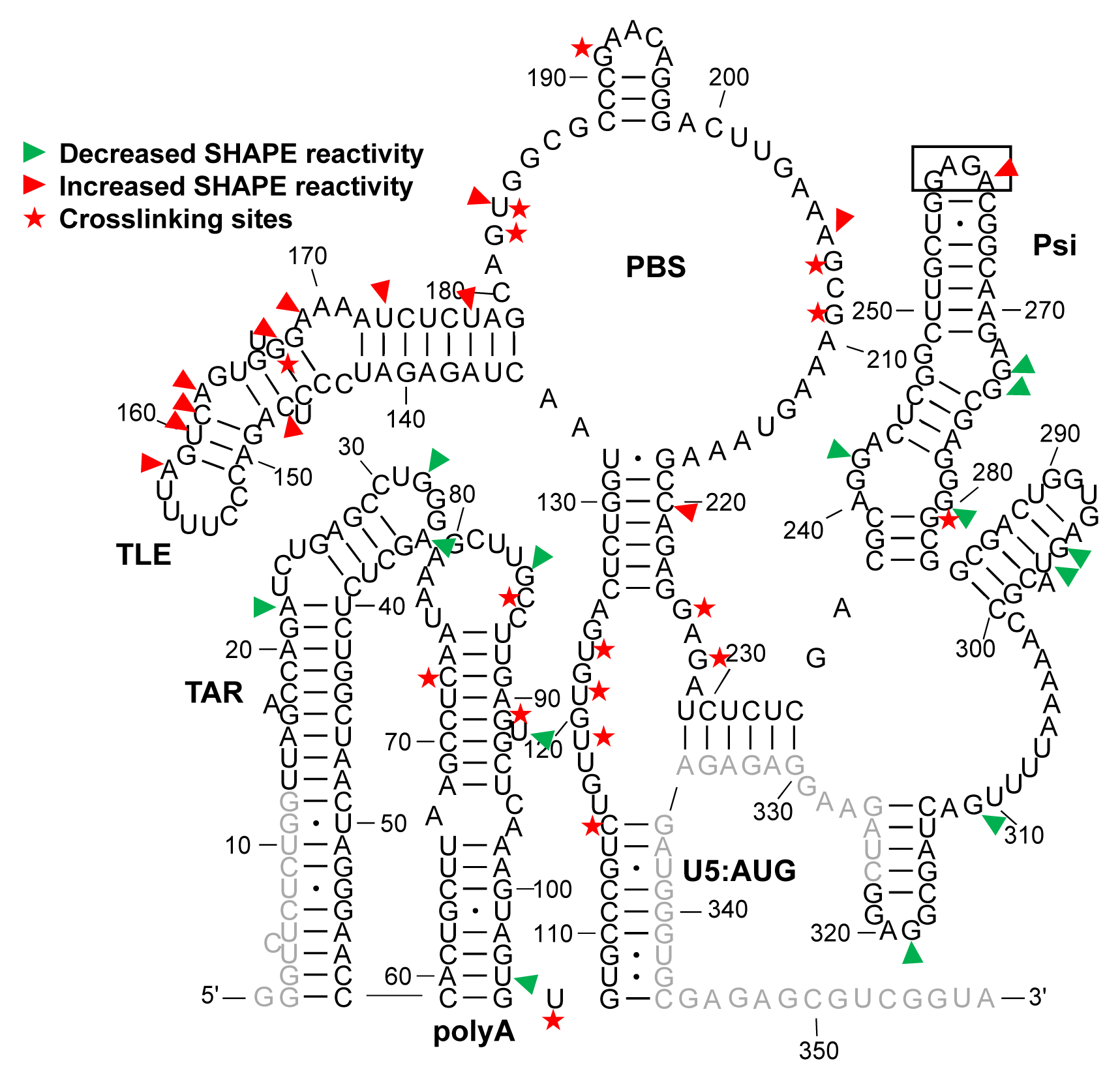

Supplement: Supplementary file 8 — Additional file 8: Figure S8. XL-SHAPE analysis of near-physiological stoichiometry of NC binding to the HIV-1 5′UTR-ΔDIS/IN/IBD complex. Sites with decreased and increased SHAPE reactivity upon protein binding are indicated by green and red arrowheads, respectively. Identified crosslinking sites are labeled with stars. All identified sites have reactivity changes of ≥ 0.3 and p < 0.05 based on unpaired, two-tailed Student’s t-tests, compared with the RNA + IN/IBD control. Results are based on the average of at least 2 independent experiments. Other information is as noted in the legend to Fig. S1. [file 12977_2021_582_MOESM8_ESM.tif]

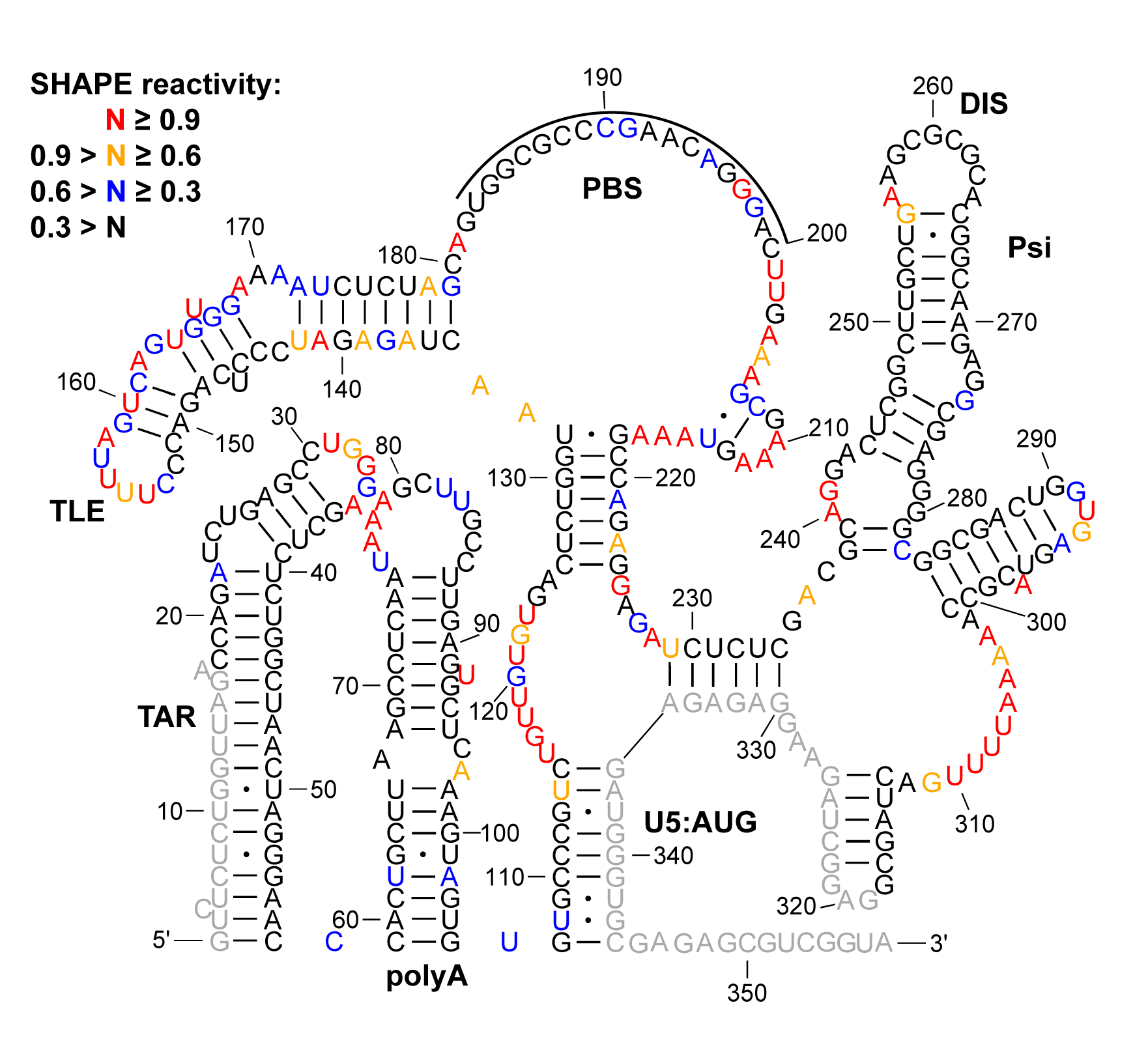

Supplement: Supplementary file 9 — Additional file 9: Figure S9. In virio SHAPE reactivity-constrained lowest energy secondary structure of the HIV-1 5′-UTR after treatment of cells with DMSO. The secondary structure model was generated by applying averaged normalized SHAPE reactivity from two independent trials as pseudo free-energy constraints. Nucleotides are colored according to SHAPE reactivity as indicated in the key. Nucleotides that could not be analyzed are shown in grey. The tRNALys,3 annealing site is indicated by a black line. [file 12977_2021_582_MOESM9_ESM.tif]
